# Astrocytes in human central nervous system diseases: a frontier for new therapies

**DOI:** 10.1038/s41392-023-01628-9

**Published:** 2023-10-13

**Authors:** Alexei Verkhratsky, Arthur Butt, Baoman Li, Peter Illes, Robert Zorec, Alexey Semyanov, Yong Tang, Michael V. Sofroniew

**Affiliations:** 1 International Joint Research Centre on Purinergic Signalling/School of Health and Rehabilitation, Chengdu University of Traditional Chinese Medicine, Chengdu, China; 2 Department of Forensic Analytical Toxicology, School of Forensic Medicine, China Medical University, Shenyang, China; 3 Faculty of Biology, Medicine and Health, The University of Manchester, Manchester, UK; 4 Achucarro Centre for Neuroscience, IKERBASQUE, Basque Foundation for Science, Bilbao, Spain; 5 Department of Stem Cell Biology, State Research Institute Centre for Innovative Medicine, LT-01102 Vilnius, Lithuania; 6 Institute of Biomedical and Biomolecular Sciences, School of Pharmacy and Biomedical Sciences, University of Portsmouth, Portsmouth, UK; 7 Rudolf Boehm Institute for Pharmacology and Toxicology, University of Leipzig, 04109 Leipzig, Germany; 8 Celica Biomedical, Lab Cell Engineering, Technology Park, 1000 Ljubljana, Slovenia; 9 Laboratory of Neuroendocrinology—Molecular Cell Physiology, Institute of Pathophysiology, University of Ljubljana, Faculty of Medicine, Ljubljana, Slovenia; 10 Department of Physiology, Jiaxing University College of Medicine, 314033 Jiaxing, China; 11 Key Laboratory of Acupuncture for Senile Disease (Chengdu University of TCM), Ministry of Education/Acupuncture and Chronobiology Key Laboratory of Sichuan Province, Chengdu, China; 12 Department of Neurobiology, David Geffen School of Medicine, University of California, Los Angeles, CA USA

**Keywords:** Neuroscience, Neurology

## Abstract

Astroglia are a broad class of neural parenchymal cells primarily dedicated to homoeostasis and defence of the central nervous system (CNS). Astroglia contribute to the pathophysiology of all neurological and neuropsychiatric disorders in ways that can be either beneficial or detrimental to disorder outcome. Pathophysiological changes in astroglia can be primary or secondary and can result in gain or loss of functions. Astroglia respond to external, non-cell autonomous signals associated with any form of CNS pathology by undergoing complex and variable changes in their structure, molecular expression, and function. In addition, internally driven, cell autonomous changes of astroglial innate properties can lead to CNS pathologies. Astroglial pathophysiology is complex, with different pathophysiological cell states and cell phenotypes that are context-specific and vary with disorder, disorder-stage, comorbidities, age, and sex. Here, we classify astroglial pathophysiology into (i) reactive astrogliosis, (ii) astroglial atrophy with loss of function, (iii) astroglial degeneration and death, and (iv) astrocytopathies characterised by aberrant forms that drive disease. We review astroglial pathophysiology across the spectrum of human CNS diseases and disorders, including neurotrauma, stroke, neuroinfection, autoimmune attack and epilepsy, as well as neurodevelopmental, neurodegenerative, metabolic and neuropsychiatric disorders. Characterising cellular and molecular mechanisms of astroglial pathophysiology represents a new frontier to identify novel therapeutic strategies.

## Neuropathology: from neuronal doctrine to a glial inclusive view

Disorders of the central nervous system (CNS), in particular those leading to cognitive deficits, are the main challenge facing medicine in the 21st century. Pathophysiologically based cures of CNS disorders do not yet exist; at best contemporary medicine is limited to symptomatic treatments. This status quo reflects the complexity of the human brain and spinal cord and lack of fundamental knowledge of multiple pathophysiological mechanisms underlying neurological disorders. Another cardinal problem faced by experimental medicine is a conspicuous translational failure of animal models of human diseases.

**Fig. 1:**
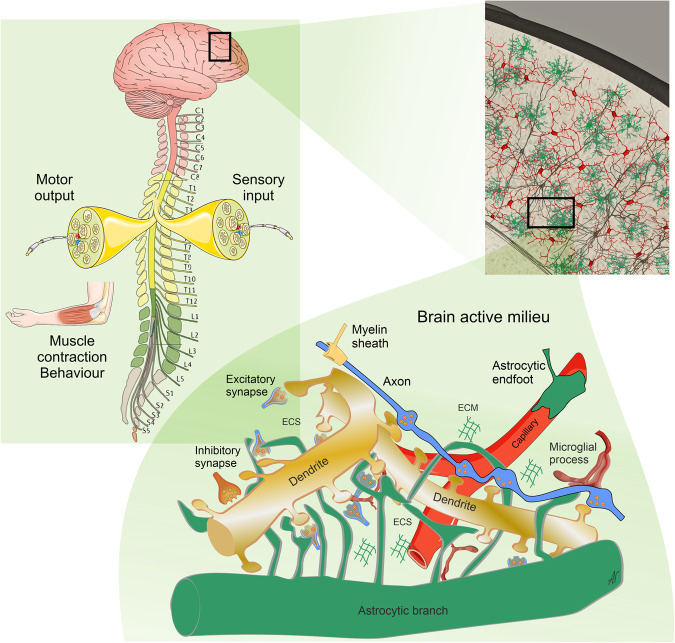
The nervous system with sensory input and motor output and multicellular active milieu of the nervous tissue. Reproduced from ref. ^6^

The human brain and spinal cord are composed of multiple cell types, including neural parenchymal cells (neurones and neuroglia) that form the active networks responsible for the functional output and a variety of supporting stromal cells (endothelial, pericytes, fibroblasts etc.) (Fig. 1).^1^ Within this active milieu, all cells are linked by numerous feed-back and feed-forward connections that stipulate coordinated interactions of all elements of the nervous tissue. At the same time different neural cells perform distinct functions: more that five hundred million years of nervous system evolution segregated neural cells into electrically excitable neurones responsible for input/output information transfer and information processing and electrically non-excitable neuroglia,^2–4^ which provide homoeostatic support and defence of the nervous tissue.^3,5–9^ Neuroglia responses and changes upon pathology are fundamental for defining the progression and outcome of neurological diseases. Neurones are highly specialised cells with limited self-protective capabilities and contribute little to adaptive nervous tissue responses to damage. When stressed, neurones limit their activity to preserve energy; if the stress continues neurones die. In contrast, when facing pathological attack, neuroglial cells upregulate neuroprotection and mount an evolutionary conserved active defensive response known as reactive gliosis. These complex adaptive glial changes counteract pathological insults. For example, reactive microglia phagocyte pathogens and cellular debris, reactive astrocytes limit damage by erecting barriers to spread of inflammation and, together with the oligodendroglial lineage cells responsible for remyelinating axons, support postlesional regeneration.

The central contribution of neuroglia to neuropathology was already recognised by Rudolf Virchow, who considered the ‘*interstitial tissue* (i.e. neuroglia) *of the brain and spinal marrow is one of the most frequent seats of morbid change*’^10^ cited from an English edition.^11^ This view on a primary pathological role of neuroglia was shared by many neuropathologists of late 19th and early 20th century.^12–17^ Unfortunately, for much of the subsequent 20th century, these concepts became superseded by the neurone doctrine, such that glial responses to CNS disorders were considered to be non-specific, stereotypic, always subsequent to primary neuronal damage and of little functional consequence. The pathological potential of neuroglia resurrected only relatively recently, while the recognition of the central role of neuroglia in neuropathology began to be universally acknowledged.^8,18–27^ Pathology of neuroglia is complex, disorder- and context-specific, and includes various forms of reactivity, atrophy, loss and gain of function. Different pathological glial phenotypes may co-exist in the same pathological process or can be associated with different stages of the disease or disorder. Glial cells to a very great extent define neuropathology, its progression and outcome: as long as glial defence prevails, the pathological process is resolved, whereas the failure of glial defence results in neuronal death and neurological deficits. Glial contributions to pathology can be primary (for example, astrocytic expression of mutant glial fibrillary acidic protein, GFAP, causing Alexander disease) or secondary, when glial cells react to pathology by mounting context-specific defensive responses. Pathological insults may also cause the death of glial cells resulting in loss of function with subsequent neuronal damage. Glial responses to pathology also reflect the degree of damage. Acute insults often cause multi-level damage including structural damage, metabolic stress and impairment of molecular homoeostasis, which trigger widespread homoeostatic failure. In chronic disorders, homoeostatic failures progress and multiply, triggering sequential and heterogeneous glial responses.

It is important to note that widespread views on neuroglia as a latent toxic cells, as dormant killers, which, when triggered, eat up healthy nervous tissue, are incorrect; as a rule it is the loss of glial supportive or protective functions which damages neurones. Similarly erroneous are widely popularised views that reactive glia polarise into simple opposing functional states that are either good or bad, neuroprotective or neurotoxic, pro-inflammatory or anti-inflammatory, A1 or A2, and M1 or M2. Such oversimplifications are incorrect and misleading (see refs. ^20,24^ for detailed discussion). At a fundamental level, most glial responses to pathology are adaptive and allostatic favouring recovery and regeneration rather than destruction. This is in keeping with the premise that ‘nothing makes sense in biology except in the light of evolution’.^28,29^ In this regard, it is notable that astrocyte reactivity is an ancient response among vertebrates and has been essentially conserved across over 100 million years of divergent mammalian evolution that separate rodents, carnivores, herbivores and primates including humans. This argues that in the context of CNS disorders, that shaped glial responses during evolution, such as responses to microbial infections and traumatic injury, astrocyte reactivity exerts essential beneficial functions. Nevertheless, astrocytes and other glia can also mediate detrimental effects in neurological disorders either through downregulation of essential functions or through gain of inappropriate functions such as promoting excess inflammation. Moreover, various pathological changes can emerge together, in sequence or in isolation being disease-, stage-, and context-specific, and are influenced by age and systemic pathologies.

## Principles of astroglial pathophysiology

### Astroglia are primary homoeostatic cells of the CNS

**Fig. 2:**
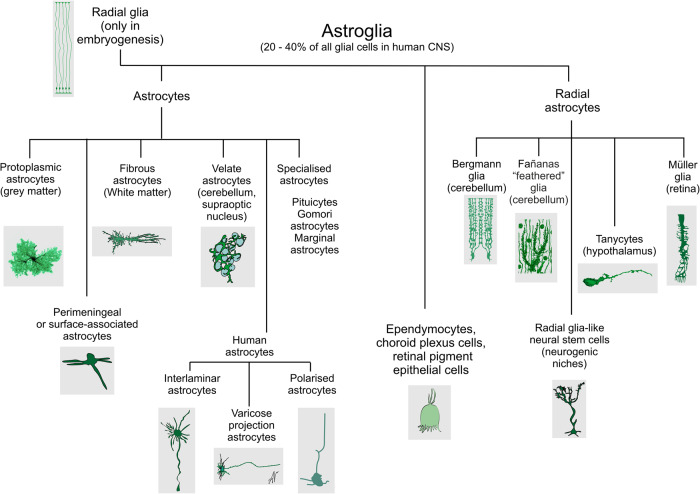
Diversity of astroglia

Astroglia (Fig. 2) are a heterogeneous class of neuroglial cells unified by their common neuroepithelial origin and their common function, which is the preservation of CNS homoeostasis. Astroglia include (i) protoplasmic astrocytes of the grey matter, (ii) fibrous astrocytes of the white matter, (iii) perimeningeal astrocytes; (iv) velate astrocytes of the cerebellum, (v) radial astrocytes (radial stem astrocytes of neurogenic niches, Müller retinal glia, cerebellar Bergmann glia and tanycytes localised mainly in the hypothalamus and in some parts of the spinal cord), (vi) pituicytes in the neurohypophysis, (vii) perivascular astrocytes, (viii) marginal astrocytes, (ix) Gomori astrocytes (rich in iron and localised in the arcuate nucleus of the hypothalamus and in the hippocampus), (x) ependymocytes, (xi) choroid plexus cells, and (xii) retinal pigment epithelial cells.^6,30,31^ Hominid primates contain several types of astroglia (interlaminar, polarised and varicose projection astrocytes) absent in the brains of all other animals.^32–35^

**Fig. 3:**
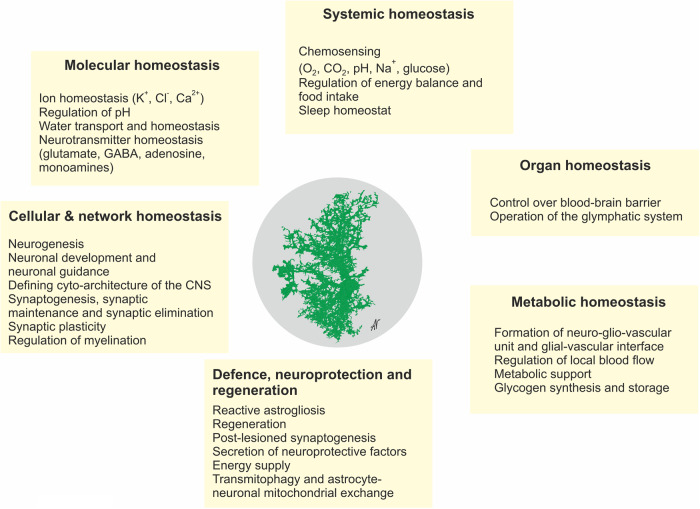
Functions of astroglia. The image of astrocyte is drawn based on 3D EM reconstruction kindly provided by Prof. Min Zhou, Ohio State University

The main role of astroglial cells in the healthy CNS (Fig. 3) is the maintenance of tissue homoeostasis at all the levels of CNS organisation, from molecular (ions, metabolites, and neurotransmitters, etc.), to network (regulation of synaptic connectivity), to organ (formation and maintenance of blood–brain barrier and glymphatic clearance system) and systemic (chemosensing blood oxygen, Na^+^, CO_2_ or glucose). Parenchymal astroglia (protoplasmic astrocytes, retinal Müller glia, cerebellar Bergmann glia) contact synapses with thin peripheral processes known as leaflets or appendages,^1,9,36,37^ which form the synaptic cradle.^38,39^ These membrane structures contain high densities of transporters that support homoeostasis in the synaptic cleft.^40^ Astroglial cells also secrete numerous factors controlling synaptogenesis, synaptic maturation and synaptic extinction.^41–44^ Many protoplasmic astrocytes in grey matter of rodents occupy individual domains and interact with neighbouring astrocytes only at the edges of these domains, with little intermingling of process among different astrocytes.^45^ Such cyto-architecture however, may not be common for all species; in particular substantial overlap of astrocytic territories was found in cortex of ferret^46^ and human.^47^ The functional logic behind this organisation is not yet understood. There is now tremendous interest in the heterogeneity of astrocytes across the CNS and there is an ongoing explosion of studies that are expanding and correlating information about structural, genetic and functional diversity of astrocytes across the healthy CNS.^9,48–56^

### Classifying astroglial pathophysiology

**Fig. 4:**
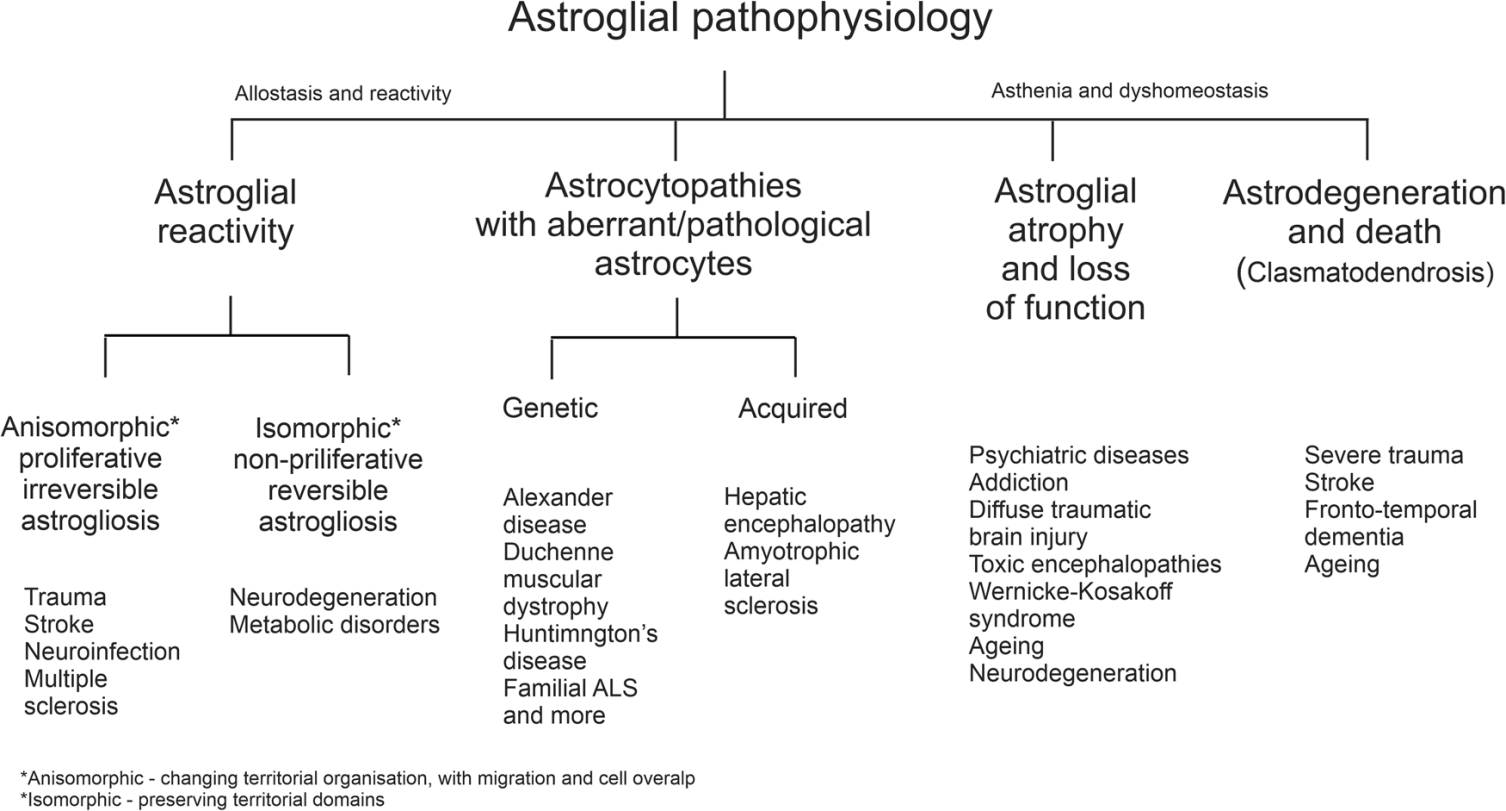
Classification of astrogliopathology

Astroglia play diverse roles in CNS disorders. Through their homoeostatic cascades, they are indispensable elements of neuroprotection that define the resilience of the nervous tissue to injury and disease. Homoeostatic systems associated with astrocytes in healthy tissue also support neuroprotection after insults, for example by supplying neurones with energy substrates in ischaemic conditions, scavenging reactive oxygen species (astroglia are the main source of glutathione), removing excess glutamate and buffering K^+^ ions, thus containing excitotoxicity, and actively taking up or detoxifying various toxic agents.^6,23,57^ Contributions of astroglia to neuropathology are however not limited to homoeostatic neuroprotection but can in some circumstances contribute to disorder progression. In pathological conditions astroglial cells undergo multiple progressive and/or regressive changes, which can to a significant extent determine the progression and outcome of neurological diseases as discussed below. Astroglial pathophysiology can be broadly classified into: (i) Astroglial reactivity or reactive astrogliosis; (ii) astroglial atrophy with loss of function; (iii) astroglial degeneration and death; and (iv) astrocytopathies with aberrant pathological astrocytes (Fig. 4).^6,23,57^

#### Astroglial reactivity or reactive astrogliosis

The concept of astrocytic reactivity or reactive astrogliosis (we shall use these two terms interchangeably) as an almost universal part of neuropathology is deeply rooted. Astrocyte responses to CNS trauma and disease have been recognised since the time of Andriezen (1895), Cajal (1913) and Alzheimer (1911). Nearly a century ago, the formation by astrocytes of a protective barrier around the fibrotic scar tissue that replaces damaged neural tissue at the lesion core after traumatic injuries was characterised by Pio del-Rio-Hortega and Wilder Penfield^58,59^ and named gliosis of astrocytes.^60^ Since that time, the terms reactive astrogliosis and astroglial reactivity (etymology: *glia* and *osis* in Greek means ‘glial process’; in Latin the suffix *-osis* acquired the additional meaning of ‘disease’ and so astrogliosis may also carry a connotation of ‘glial disorder’) have become widely and interchangeably used to describe astroglial responses to pathology.^20^

Astroglial reactivity can now be defined as an evolutionarily conserved, graded, and multi-stage primarily defensive reaction of astrocytes to neuropathology.^20^ Thus, by definition, astroglial reactivity is always secondary, being a response of astroglial cells to a pathological process. Astrocytic reactivity reflects activation of complex molecularly defined programmes which define remodelling of biochemical, morphological, metabolic, and physiological properties of astroglia leading to an upregulation or loss of homoeostatic cascades, or in gain of new protective or regenerative functions.^20^ Astrocytic reactivity is highly context dependent and is manifested by many different reactive phenotypes or transient states. Astrocytic transcriptomes and molecular signatures in various neurological diseases are highly diverse, again highlighting the heterogeneity of this process.^61–66^

**Fig. 5:**
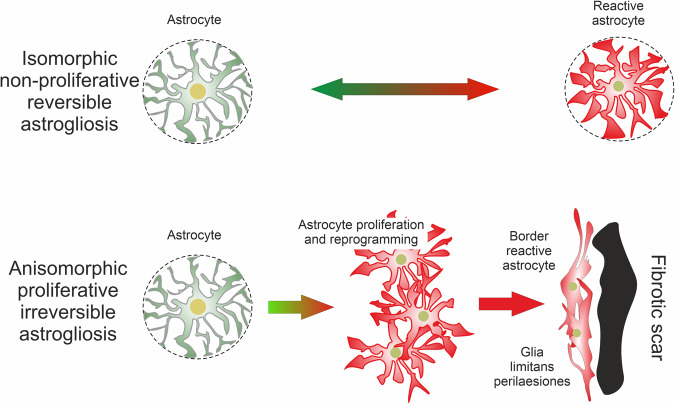
Classification of reactive astrogliosis

Astrocyte reactivity phenotypes can at present be broadly classified into two major categories of (i) non-proliferative astrogliosis, which is isomorphic with mainly preserved domain organisation; and (ii) proliferative astrogliosis, which is anisomorphic with loss of domain, substantial structural reorganisation and can either be diffuse or can result in the formation of new compact ‘limitans’ borders around overt fibrotic tissue lesions (Fig. 5). Notably, within these broad categories, reactive astrocytes can exhibit substantial differences in molecular signatures and functional states that can vary with tissue region and disorder context as discussed below. Many details of reactive astrocytic remodelling remain to be revealed and characterised.

*(i) Non-proliferative astrogliosis*. In the healthy adult CNS, astrocytes rarely divide and are essentially post-mitotic.^67^ Non-proliferative astrogliosis typically occurs in neural tissue that is responding to a pathology but is not overtly damaged and retains its basic tissue architecture, for example (i) during diffuse neuroinflammation caused by peripheral exposure to microbial antigens such as lipopolysaccharide (LPS),^68,69^ (ii) in tissue regions that are at a distance from focal lesions (diaschisis) caused by stroke, trauma or autoimmune attack,^8,70^ or (iii) in tissue undergoing neurodegenerative changes.^71^ Non-proliferative reactive astrogliosis can vary in intensity but is isomorphic such that the astrocytes mainly retain the discrete, non-overlapping cellular domains found in the healthy grey matter but with variable degrees of cellular hypertrophy and reorganisation of their processes.^72^ These astrocytes maintain their interactions with local neurones, synapses, oligodendrocytes and vascular cells but may alter these interactions in accordance with context-specific reactive changes in molecular expression and functions.^65,66^ Notably, molecular changes exhibited by non-proliferative reactive astrocytes can be fully reversible over time after single exposures for example to neuroinflammation caused by LPS.^68^

*(ii) Proliferative astrogliosis.* Astrocytes can re-enter the cell cycle and proliferate in response to overt tissue damage such as is caused by stroke, severe trauma, infection, foreign bodies (including medical implants), autoimmune inflammation, neoplasm or severe neurodegeneration.^19,67,73,74^ Newly proliferated reactive astrocytes form borders that separate damaged, inflamed, and fibrotic tissue from adjacent viable neural tissue, and during this border formation newly proliferated astrocytes become transcriptionally reprogrammed to adopt new cellular interactions with non-neural cells.^19,65^ Although most border-forming reactive astrocytes in narrow zones immediately abutting tissue lesions are newly proliferated, proliferation drops off rapidly with increased distance from lesions, which are surrounded by large areas of intermingled proliferative and non-proliferative reactive astrocytes as well.^67,75^ Most border forming astrocytes derive from local astrocytes,^67^ with a small contribution from proliferation of adult OPC.^76,77^ Proliferation of astrocytes is an indispensable part of reactive astrogliosis, and suppression of proliferation exacerbates damage and delays wound closure. There is a widespread and popular belief regarding reactive astrogliosis and astrocytic perilesional border as a harmful reaction that limits regenerative capacity of the nervous tissue. This is an incorrect view; reactive astrogliosis is fundamentally protective and is indispensable not only for wound closure and formation of a barrier separating fibrotic scar from the healthy tissue but also for postlesional regeneration. Suppression of normally occurring reactive astrogliosis worsens neurological outcome and inhibits postlesional plasticity, regeneration and repair, as well as a restoration of the blood-brain barrier, which all determine functional tissue remodelling and post-traumatic rehabilitation.^65,78–86^

Thus, at present, non-proliferative and proliferative astrocyte reactivity represent two broad categories that can be readily differentiated and are associated with diverse molecular and functional differences. Notably, neither of these categories should be regarded as homogenous or stereotypic, and differences among reactive astrocytes within them are being identified. There is accumulating evidence for diverse changes in molecular expression of reactive astrocytes in different types and different severities of tissue pathology,^62,65,68,69^ but there is not yet a good synthesis of how molecular, metabolic,^87^ structural, and functional changes relate to one another to generate precisely definable phenotypes.

*(iii) Scar tissue in the CNS is fibrotic and not glial.* In the CNS as in any other tissue, focal tissue damage is closed through rapid proliferation of stromal cells and formation of a fibrotic scar, which is the replacement of the lost parenchymal cells (i.e. neurones and neuroglia) with stromal cells (fibroblasts and pericytes). In this regard, CNS tissue is not different from any other tissue. It is primarily the stromal cells that produce extracellular matrix and form fibrotic scar tissue. The CNS differs in that the formation of this fibrotic scar develops in parallel and in coordination with reactive astrogliosis, which generates a perilesional astrocyte border that has similarities in appearance and function (and in molecular mechanisms involved) to the glia limitans formed by perimeningeal astrocytes that interface with stromal cells of the meninges around the entire CNS.^19,88^ Like perimeningeal astrocyte borders, the newly formed perilesional astrocyte borders also serve to separate neural tissue from non-neural tissue.^85^ The main scar-forming cells in the CNS are perivascular fibroblasts^89^ and type A pericytes,^90^ which produce the fibrotic extracellular matrix that cements the scar.^91^ There is a long history of referring to the glia that surround CNS as ‘glial scars’ or as ‘astrocyte scars’, but multiple lines of evidence challenge this usage. In no other organ are parenchymal cells that proliferate after injury referred to as scar tissue. Astrocytes are neural parenchymal cells that proliferate after injury to replace lost neural tissue, form borders, and protect and preserve neural parenchyma in different disorder contexts as discussed in multiple places in this article. We suggest that it is time to stop referring to these structures as ‘glial scars’ or ‘astrocyte scars’ and instead refer to them as ‘astrocyte borders’ or ‘glial borders’.^19,88^

*(iv) Markers of astrocyte reactivity.* Morphological changes of reactive astrocytes have long been recognised not only after traumatic injuries but also in many other pathological contexts.^23^ In addition, for many decades increased immunostaining with antibodies against glial fibrillary acidic protein (GFAP) has been regarded as a universal molecular marker of astrocyte reactivity. It must be noted, however, that increased GFAP expression and GFAP-positive astrocytic profiles are not always associated with pathology. Physiological stimulation (such as, for example, physical activity, environmental enrichment, exposure to various diets or even circadian rhythmicity) may significantly change GFAP levels and morphometric parameters of GFAP-positive cellular profiles.^92–94^ There is now strong interest in identifying molecular markers associated with astrocyte reactivity. As noted above, astrocyte reactivity is associated with diverse changes in molecular expression that can vary from mild to pronounced and that are highly context dependent. Increases in the expression of various molecules that have been noted in reactive astrocytes across multiple contexts include GFAP, vimentin, nestin (probably labelling proliferating astrocytes), synemin plectin, α-crystallin B chain, monoaminoxodase-B (MAO-B), heat shock factor binding protein 1, complement C3, lipocalin 2, C-X-C motif chemokine ligand 10, SerpinA3N, LCN2 and others,^20,95–103^ but it important to note that no single molecular marker (including GFAP) is an absolute, required and sufficient, indicator of astrocyte reactivity, and no molecular markers have yet been identified that reliably distinguish amongst different reactive astrocyte phenotypes. In the future, rather than look for additional global markers of astrogliosis, it will likely be more useful to look for molecules upregulated by astrocytes in specific contexts and that are associated with specific functions or effects of astrogliosis.

To summarise, normally occurring reactive astrogliosis is in the first instance an intrinsic and evolutionary conserved set of diverse astrocyte responses that are aimed at neuroprotection, maintenance of tissue homoeostasis and preservation of nervous tissue integrity.

#### Astroglial atrophy and loss of function

A widespread class of astrocytic changes observed in many neurological diseases and in the majority of neuropsychiatric disorders are represented by structural atrophy and functional asthenia and manifested in the loss of key homoeostatic functions, such as for example glutamate clearance. This atrophy and loss of function are often a primary cause of neuropathology, as for example is caused by substantial decreases in expression and function of glutamate transporters in Wernicke-Korsakoff encephalopathy or toxic brain damage.^104,105^ Functional asthenia of astrocytes is frequently associated with morphological atrophy, decrease in territorial domain and associated reduction in astrocytic synaptic coverage. These morphological changes decrease astrocytic presence in the neuropil and hence diminish their homoeostatic support of the nervous tissue. In particular, morphological atrophy of astrocytes is prominently presented in neuropsychiatric diseases including mood disorders, post-traumatic stress disorders, addiction, and in some forms of autistic spectrum disorders.^106–111^ Similarly, morphological atrophy and functional asthenia of astrocytes contribute to the pathophysiology of various neurodegenerative diseases such as Alzheimer’s disease (AD), Parkinson’s disease (PD) and Huntington’s disease (HD).^112–117^ Arguably, atrophic astrocytes cannot properly support synaptic transmission, which results in cognitive and psychiatric syndromes.

#### Astroglial degeneration and death (Clasmatodendrosis)

**Fig. 6:**
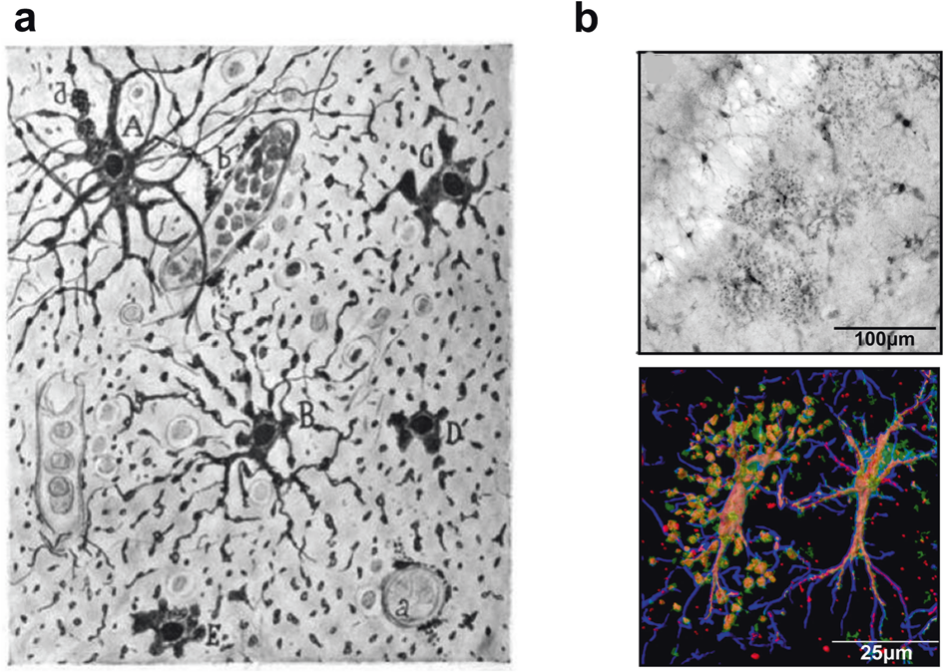
Clasmatodendrosis of astrocytes. **a** Clasmatodendrosis as seen and drawn by Ramon y Cajal.^118^ A. Cell with preserved processes. B. Astrocyte with fragmentations. C, D, E. Astrocyte with disrupted cytoplasmic expansions, but with preservation of perikaryon. a. capillary. b. disaggregated end feet. **b** Clasmatodendrosis of astrocytes in the aged brain of mouse (stratum radiatum of dorsal hippocampus). Upper panel Astrocytes with distinctive enlarged soma and vacuolisation of processes distinctive to clasmatodendrosis for a representative cluster of astrocytes. Lower panel: Imaris surface render of a confocal z-stack of GFAP (blue), S100β (green), and Vimentin (Red) demonstrates an astrocyte with clasmatodendrosis (left) showing the co-localisation of S100β+Vimentin+ beads along GFAP+ processes, and a reactive astrocyte with non-degenerative morphology adjacent to it (right). Reproduced from ref. ^120^

Many neuropathologies, including for example traumatic lesions, stroke or age-dependent diseases are associated with direct damage of astrocytes, causing their degeneration and death. Morphologically, degeneration and death of astrocytes are known as *clasmatodendrosis* (from Greek ‘κλάσμα’, fragment, ‘δένδρον’, tree, ‘ωσις’, process), described, for the first time, by Alois Alzheimer, who found disintegrating, fragmented processes of astrocytes and oligodendrocytes in epilepsy, neurosyphilis and dementia.^14^ Ramon y Cajal considered clasmatodendrosis as an early post-mortem artefact.^118^ Nonetheless, many studies demonstrated clasmatodendrosis as an outcome of pathologies, which occur in the pre-mortem tissue.^119^ Clasmatodendrosis (as the name suggests) is manifested by fragmentation of astrocytic processes, disappearance of distal processes, together with swelling and vacuolation of the cell body (Fig. 6). Clasmatodendrosis was described in traumatic brain injury, cerebral ischaemia, post-stroke dementia, status epilepticus, demyelinating diseases, cerebral oedema, toxic encephalopathies, small-vessel disease and neuroinfection.^119,120^ Prominent astrocyte degeneration and fragmentation appears as a common feature of fronto-temporal dementia; notably astrocytic degeneration correlates with the severity of the disease.^121^ Clasmatodendrosis of astrocytes was also found in the white matter of some post-mortem brains of AD and cerebrovascular pathology.^122^ All in all, the degree to which degeneration and loss of astrocytes may contribute to different neurological disorders is understudied and deserves more attention.

#### Astrocytopathies with aberrant pathological astrocytes

Aberrant astrocytes that may act as instigators and propagators of neuropathology have been described in several diseases; these aberrant forms are covered under the umbrella term of astrocytopathies.^18,23,123^ The prototypical example of a genetic primary astrocytopathy is Alexander disease, a leukomalacia in which astrocytes express mutant GFAP, although how the expression of this mutant gene translates into severe damage of the white matter remains unknown.^124,125^ Another example of genetic primary astrocytopathy is the Duchenne muscular dystrophy (DMD) caused by mutations in the gene encoding dystrophin, which in the CNS is present almost exclusively in astrocytes. Expression of this mutated gene greatly reduces expression and operation of astrocytic glutamate transporters, thus leading to an excitotoxicity linked to psychosocial abnormalities and impaired cognition.^126,127^ Familial genetic mutations and polymorphisms can also alter astrocyte functions in ways that contribute to disorder progression as noted in Huntington’s disease,^117,128^ familial amyotrophic lateral sclerosis (ALS) mutations,^129,130^ or familial AD mutations.^131^ In addition, gene polymorphisms can alter astrocyte responses to pathologies and contribute to disorder progression, as for example with APOE polymorphisms in AD or traumatic injuries,^132–135^ or with CD38 polymorphisms in PD.^136^ In addition, acquired astrocytopathies are prominent in hepatic encephalopathy (in which astrocytes lose their homoeostatic capabilities^137^) and in neuromyelitis optica, in which the astrocyte protein, AQP4 is the subject of autoimmune attack, leading to dysfunction and death of astrocytes resulting in pronounced inflammation and degeneration of neural tissue.^85,138^ Aberrant astrocytes expressing markers of both astrocyte and microglia have been detected in ALS, in stroke and in dementia with Lewy bodies.^139,140^

### Astroglial detrimental effects through loss or gain of functions

**Fig. 7:**
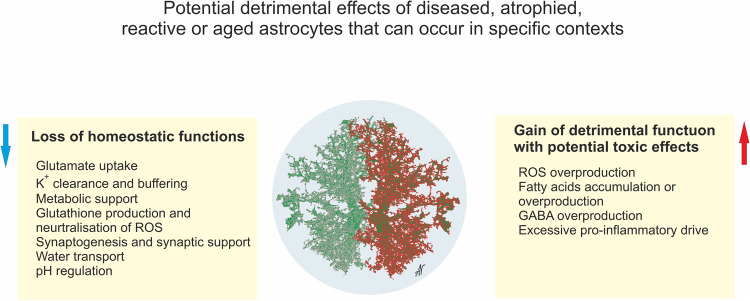
Potential detrimental effects of diseased, atrophied, reactive or aged astrocytes that can occur in specific contexts. See text for further explanation

There is mounting interest in how astrocytes that are reactive or diseased might have detrimental effects on the outcome of disorders. Although astrocyte reactivity is likely in the first instance to be targeted at maintaining CNS homoeostasis and circuit functions, various mechanisms could alter astrocyte functions in potentially detrimental ways, including (i) persistent reactivity may contribute to chronic neurodegenerative disorders or chronic inflammation, (ii) ageing and cellular senescence which may alter and reduce astrocyte functional capacities, or (iii) genetic mutations in diseased astrocytes or polymorphisms may alter normal astrocyte functions or responses. Such mechanisms could detrimentally impair astrocyte functions either through the loss or down-regulation of essential homoeostatic functions or through the gain of detrimental effects, or combinations of both. The potential loss of essential homoeostatic astrocyte functions includes (i) down-regulation of glutamate uptake that disturbs circuit function and increases excitotoxic potential^141–143^; deficient glutamate clearance is the primary element of several severe neurodegenerative diseases such as for example in ALS,^144,145^ and Wernicke’s encephalopathy^146^; (ii) down-regulation of K^+^ buffering resulting a neuronal hyper-excitability that disturbs circuit function and increases excitotoxic potential^128^; (iii) disruption of metabolic support of neurones and oligodendrocytes^147,148^; (iv) down-regulation of production of glutathione scavengers of cytotoxic reactive oxygen species (ROS)^149,150^; (v) reduced synapse support in ageing^151^ (Fig. 7). Gain of potential detrimental functions or effects could include: (i) increased pro-inflammatory signalling which at first is adaptive but can also be chronic and contribute to excess inflammation and degeneration^152,153^; (ii) increased GABA production resulting in disruption of circuit functions^154,155^; (iii) increased production of cytotoxic ROS^155,156^; (iv) increased accumulation or production of potentially toxic saturated very long chain fatty acids^153,157^ (Fig. 7). It has to be noted however that gain of function may also develop in parallel with loss of function, with reactive astrocytes for example losing their ability to clear glutamate in the context of motor neurone diseases.^158^ Similarly, modifications of astrocyte sphingolipid metabolism in reactive astrocytes affect their metabolic support of neurones and have indirect effects in this manner.

Notably, many if not most, gains of potentially detrimental effects come about through the loss or malfunction of physiological astrocytic processes, rather than through the activation of pre-programmed and intentionally ‘toxic’ mechanisms. For example, transcriptional regulatory evaluations found no evidence for a programme of gain of function changes common across different degenerative disorders.^65^ Thus, gain of detrimental effects is likely to be context-dependent and unique to specific situations and effective treatment will require understanding the distinctive situations under which they arise. It deserves emphasis that to date there is no rigorous evidence for a programmed ‘neurotoxic’ astrocyte phenotype that is somehow activated in a common manner across multiple disorders and represents a ‘universal’ target, in spite of certain claims to this effect. Understanding the molecular and cellular mechanisms underlying the loss or gain of disorder-related detrimental effects mediated by astrocytes in different contexts has the potential to identify new treatment strategies for a wide variety of CNS disorders.

### Conclusions

Astrogliopathology is complex, heterogeneous and context dependent with regard to disorder, disorder-stage, comorbidities, age, and sex. Reactive astrogliosis is in the first instance an evolutionary conserved protective response of the nervous tissue. In traumatic injuries (neurotrauma, neuroinfection, stroke or immune attack), irreversible proliferative astrogliosis is essential for wound closure and formation of a glial border that separates fibrotic scar tissue from the healthy neural tissue and promotes postlesional regeneration. Non-proliferative astrogliosis occurs in neural tissue responding to surrounding tissue pathology; astrocytes are not overtly damaged and maintain their interactions with local neurones, synapses, oligodendrocytes and vascular cells.

Although astrocyte reactivity is likely to be aimed at maintaining CNS homoeostasis and circuit functions, various mechanisms could alter astrocyte functions in potentially detrimental ways through the loss or down-regulation of essential homoeostatic astrocyte functions or through the gain of detrimental effects. Of note, the decrease in homoeostatic capacity of astrocytes seems to be the prevailing mechanism across various neuropathologies. In addition, cell autonomous primary astrocytopathies can give rise to aberrant astrocytes which drive neuropathological progression, and there is now mounting interest in how astrocytes that are reactive or diseased might have detrimental effects on the outcome of disorders.

## Astrocytes control CNS damage: neurotrauma, stroke, neuroinfection and autoimmune attack

### Neurotrauma

#### Acute focal traumatic brain injury

Acute focal traumatic brain injury (TBI) can be caused by penetrating lesions to the brain parenchyma that trigger cell death and haemorrhage, or by external force impact against the head resulting in brain contusion with intra-parenchymal haemorrhage, cell death and axonal damage. Fractures of the skull, vasogenic oedema, epi-, or subdural or intracerebral haematoma are frequently accompanied by focal TBI.^159^ The size and localisation of these focal injuries can vary widely, and this variability defines immediate and long-term neurological consequences.

**Fig. 8:**
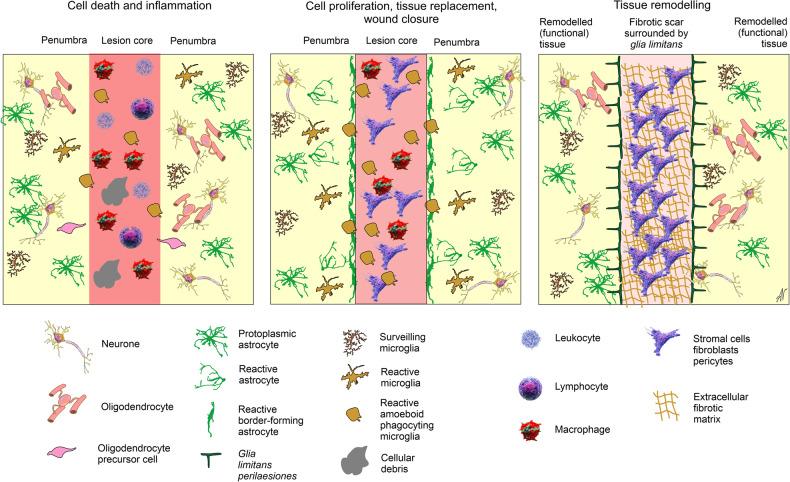
Stages of neuroinflammation and scar formation following traumatic brain injury. See text for explanation. Modified from ref. ^6^

The response to acute focal TBI with tissue damage develops in the following phases: (i) cell death and inflammation, (ii) cell proliferation, tissue replacement with fibrotic scar and wound closure and (iii) tissue remodelling and neuroplasticity aimed at restoration and functional compensation^26,160^ (Fig. 8). The first phase of CNS response to acute focal TBI is the disruption of the blood-brain barrier and traumatic injury of parenchymal cells. Mechanical forces trigger primary cell death, cellular lysis and cytotoxic oedema.^161^ Overactivation of ionotropic receptors as well as mechanoporation of cellular membranes^162^ lead to a massive Ca^2+^ entry that mediates cellular death.^163^ At the lesion core therefore, all neural cells die by necrosis, which leads to a massive release of damage-associated molecular patterns (DAMPs), including release of glutamate and ATP, which propagate excitotoxicity.^164–166^ Breach of blood-brain barrier results in the infiltration of blood-borne cells (erythrocytes, leucocytes, macrophages, and platelets) and molecules (such as fibrin, fibrinogen, collagen, or albumin). Invading white blood cells secrete pro-inflammatory factors, which trigger reactive response of neural parenchymal cells, most notably reactive astrogliosis and microgliosis. Similarly, fibrin, fibrinogen, collagen and other blood-derived molecules, together with DAMPs released from dying cells signal to neuroglia and instigate reactive gliosis.^19,167,168^

Reactive gliosis, which starts within hours after the lesion, is a key and CNS-specific response to the TBI. The first responding cells are microglia and adult oligodendrocyte precursor cells (OPC), which migrate towards the lesion, with astrocyte responses to follow. There is a gradient of reactive morphotypes, with the most reactive cells (border-forming astrocytes and amoeboid microglia) concentrating at the lesion perimeter, or even entering (reactive microglia) the lesion core.^75,169,170^ Reactive microglia first position themselves between the infiltrating lymphocytes and newly proliferating reactive astrocytes that will form the perilesional border of astrocytes.^171–173^ Less prominent reactive morphotypes represented by polarised cells, which extend their processes toward the lesion area, are positioned more distantly, whereas healthy looking neuroglia demarcate the undamaged tissue. Adult OPC also undergo several forms of peculiar reactivity: some of them transform form into bipolar cells resembling foetal OPC, which migrate towards the lesion; adult OPC located distantly become hypertrophic with larger somata and more complex processes.^77,174,175^ Of note, GFAP expressed by reactive astrocytes following TBI, or released by damaged or dead astrocytes, can be also detected in the blood and spinal fluid, and the levels of GFAP in these fluids can reflect the severity of trauma and can be used as a diagnostic clinical tool.^176^

The second phase of the nervous tissue response is cell proliferation, and this is manifested by proliferation of fibroblasts and pericytes, which form extracellular matrix to produce the fibrotic scar^177,178^ that rapidly replaces lost neural parenchyma. In addition, reactive astrocytes at the perimeter of the fibrotic scar proliferate to form perilesional barrier that protects the adjacent viable nervous tissue. In mice, the peak in glial proliferation is observed during days 2 to 7 days after trauma, and subsequently proliferation gradually subsides.^67,179^ This second phase of acute focal TBI is complete about a month after the insult, when the mature lesion, composed of the central fibrotic scar (fully devoid of neural elements) and the surrounding astroglial limitans border have formed.^26,160^ Subsequently, the final phase of tissue remodelling plasticity starts; this phase may last for months and years during which the reshaping of neuronal ensembles provides for functional recovery. Perilesional astrocytic border (again contrary to a widespread beliefs) is permissive for axonal outgrowth and tissue recovery; ablation of glial limitans prevents functional rehabilitation.^78,86,180^ Neurotrauma boosts neurogenesis and migration of neuroblasts into the perilesional penumbra, where they arguably contribute to the neuronal circuitry repair.^181^

#### Diffuse traumatic brain injury

Diffuse (also known as mild) TBI results from the action of acceleration or deceleration forces on the head, which leads to a strain and concussion of the brain tissue. These mechanical forces induce sub-lethal damage to the cellular elements and may cause local disruption of the blood–brain barrier, with subsequent extravasation of blood cell and blood-born factors triggering focal inflammation.^182^ Histopathology of diffuse brain injury also includes diffuse axonal damage, axonal swelling, and disconnection.^183^ In about 50% of cases diffuse traumatic brain injury is complicated by long-term neurological consequences including cognitive decline, sleep disturbances and depression.^184^ Astrocytic reactivity in diffuse brain injury is quite distinct from full blown reactive astrogliosis in focal TBI. In cortex for example, astrocytes in response to diffuse injury up-regulate GFAP and become mildly hypertrophic, however they retain their territorial domain and generally do not proliferate. At the same time these astrocytes reduce expression of several key homoeostatic proteins such as glutamate transporters, glutamine synthetase, K_ir_4.1 inward rectifying channels involved in K^+^ buffering, and connexion 43 responsible for syncytial coupling.^185^ These asthenic astrocytes emerge shortly after the trauma and remain in the damaged area for months after injury. These malfunctional astrocytes are characterised by a prominent functional asthenia and lose key homoeostatic proteins, such as glutamate transporters, K_ir_4.1 channels, glutamine synthetase and gap junctional protein connexin 43, which results in an uncoupling of astrocytic syncytia.^185^ The aberrant asthenic astrocytes remain in the brain for months after the initial injury arguably delaying the recovery.^185,186^

#### Chronic traumatic encephalopathy

Chronic traumatic encephalopathy (CTE), which results from multiple and repeated mild traumatic injury (experienced for example by professional footballers, hockey players or boxers—hence its name ‘dementia pugilistica’^187^; *pugilator* - boxer in Latin), is a progressive neurodegenerative condition characterised by memory deficits, disorientation, confusion, aggression, and improper behaviours. In this pathology astrocytes undergo mild reactive changes and often develop astro-tauopathy (see below). Prominent clasmatodendrosis of astrocytes was observed in ~70% of post-mortem brains of patients diagnosed with CTE.^188^ CTE can also lead to atypical astrocyte responses that contribute to recurrent seizures.^185^ CTE is of steadily growing interest as a risk factor for various neurodegenerative disorders.

#### Spinal cord injury

For many decades, since the 1940s, based purely on correlative observations, reactive astrocytes that surround tissue lesions after traumatic injuries or stroke were regarded as ‘glial scars’ that were the primary cause for the failure of axon regeneration and functional recovery after spinal cord injury (SCI) and other causes of axotomy in the CNS.^189^ Recent studies overturned this long-standing dogma by showing that multiple experimental approaches to removing or genetically attenuating the astrocyte borders that form around lesions all failed to result in spontaneous axon regeneration.^86^ Moreover, other experimental approaches showed that substantial axon regeneration through lesions can be achieved by providing growth stimulating and chemoattractive factors, and this stimulated growth is attenuated (and not augmented) by disrupting astrocyte borders.^86,190^ There are now multiple lines of evidence that the failure of axon regeneration after SCI and other CNS injuries is due to multiple factors, including the failure of mature CNS neurones to reactivate and sustain developmental growth programmes, combined with a lack of appropriate chemoattraction.^86^^,^^189–193^ Indeed, there is increasing evidence that astrocyte borders around lesions can in fact support regrowing axons,^86,190,194^ suggesting that it is time to retire the term ‘glial scar’ when referring to astroglial borders around CNS tissue lesions.^19,189^

### Ischaemia and stroke

Brain ischaemia can be caused by a systemic fall in blood supply (for example due to heart failure) or by occlusion of blood vessels through gradual thrombus formation or by acute embolism. Ischaemia can also be caused by the rupture of intracranial vessels, resulting in intracerebral haemorrhage. Ischaemia can be global or focal and acute or chronic. Global ischaemia causes widespread damage and cell death; about 10 minutes of global ischaemia usually is lethal. Focal ischaemia can trigger local damage to the nervous tissue ranging from cell death at the ischaemic core and various degrees of functional cellular deficits in the ischaemic penumbra. Ischaemia that causes acute functional deficits is commonly referred to as a stroke. The pathophysiology of ischaemic stroke is defined by the limitation of oxygen supply (hypoxia or anoxia), and restricted supply of metabolic substrates. The degree of damage is directly proportional to the degree of blood flow restriction. Decrease of blood flow below 1 ml/g/min causes total cellular death in the affected area. Cell death is a consequence of the decrease in ATP production which rapidly compromises ion (mainly due to halting Na^+^-K^+^ pump) and acid-base homoeostasis.^195–197^ At the cellular level, this translates into a massive increase in cytoplasmic Na^+^ concentration, membrane depolarisation and opening of voltage-gated Ca^2+^ channels, which in turn promotes massive release of glutamate that results in even large depolarisation enlarging Na^+^ and Ca^2+^ influx thus completing the vicious circle of excitotoxic damage, as Ca^2+^ overload triggers necrotic cell death.^163,164,198,199^ Breakdown of ion homoeostasis ion gradients, uncontrolled neurotransmitter release, oedema and mitochondrial failure are thus the key mechanisms behind pathophysiology of the ischaemic stroke.

The ischaemic core, where all neural cells are dead, is surrounded by the penumbra, in which cells are functionally compromised, but still surviving and can potentially be rescued. ATP production in the cells in the penumbra is reduced by ~50–70%, which supports some semblance of ion homoeostasis. At the same time, cells in the penumbra are subjected to periodical transient ischaemic depolarisations,^200^ mechanisms of which are essentially similar to spreading depression. Survival or death of neural cells in the penumbra correlates with the frequency of these transient ischaemic depolarisations. In essence, the balance between survival and death depends on neuroprotection and energy state of the tissue.^201^ This neuroprotection and support are mainly provided by astrocytes. First, astrocytes protect neurones in the penumbra, and second, after the end of infarct expansion, astrocytes form a peri-infarct barrier very similar to the perilesional barrier in the TBI.

In general, astrocytes resist ischaemic attacks better than neurones. Oxygen-glucose deprivation in cell cultures kills all neurones within an hour, whereas astrocytes survive for several hours more.^202,203^ In vivo, astrocytes are more sensitive to periods of ischaemia, although they still tolerate them better than neurones.^200,204^ Astrocytes survive even better in the penumbra, which is exposed to a lesser ischaemia. Astrocytes can at least temporarily switch to the glycolytic pathway to support their own energetics, and can use their glycogen pool (of which they are the sole possessors in the CNS^205,206^) to produce lactate and support energy substrate-deprived neurones.^207^ It must be remembered however, that an increase in lactate synthesis results in acidosis, which may severely damage astrocytes, and by proxy neurones. Hence, delivering glucose to the stroke affected brains exacerbates the infarction.^208,209^ In addition to providing energy support, astrocytes buffer glutamate overload through their glutamate transporters: ablation of the latter exacerbates ischaemic damage.^210^ Furthermore astrocytes are key elements for anti-oxidative defence being the main scavengers of reactive oxygen species.^211^

**Fig. 9:**
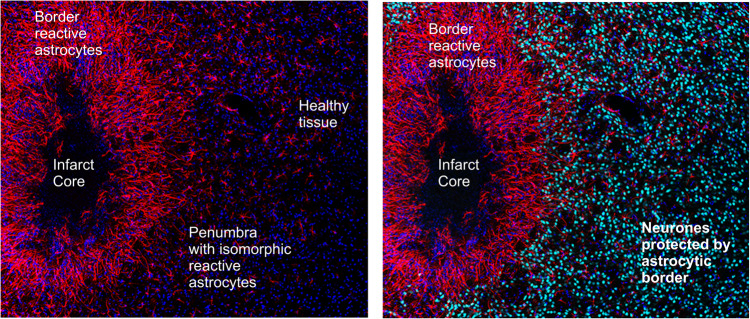
Reactive astrogliosis and protective astrocyte border formation in experimental stroke. Images show an ischaemic infarct in mouse striatum at 14 days after injection of the vasoconstrictive agent, L-NIO (N^5^-(1-Iminoethyl)-L-ornithine) in a manner similar to that described previously.^78^ Immunohistochemistry shows GFAP-expressing astrocytes stained red and NeuN-expressing neurones stained light blue. The left image shows the border of newly-proliferated reactive astrocytes that surround the fibrotic tissue (unstained) of the infarct core and isolate it from adjacent neural tissue. The right image shows a normal density of healthy neurones immediately adjacent to the protective astrocyte border. Images are courtesy of Dr. Shinong Wang and Dr. Yan Ao

Astrocytes also protect the brain tissue through reactive astrogliosis. The stroke is invariably accompanied by the breach of the blood-brain barrier and infiltration of blood-borne elements into the brain parenchyma. This, together with cell death leads to a massive release of release of DAMPs including ATP, heat shock proteins, peroxiredoxins, and many others.^212–214^ These DAMPs trigger gliotic responses, initially represented by migration of microglia and OPCs as described previously, and second, after the end of infarct expansion, reactive astrogliosis. The infarct core is infiltrated with macrophages, dendritic cells and reactive microglia, which by combined effort, clear cellular debris. Reactive astrocytes proliferate and form, around the inflammatory cells and fibrosis of the infarct core, a barrier that protects the adjacent neural tissue (Fig. 9).^160^ A gradient of astrocytes at different reactive states is observed in a penumbra zone towards the healthy tissue, very much similar to that observed in the TBI and SCI.^78,215,216^ With time the dead tissue is replaced with fibrotic scar surrounded by glia limitans barrier that fences the healthy tissue and assists post-stroke regeneration.^78^ Grafts of neural progenitors that generate astrocytes can reduce stroke lesion volumes and promote repair.^78,217^ Notably, some reactive astrocytes, in the context of ischaemic brain damage, become actively phagocytic, thus contributing to the removal of damaged cells and assisting post-stroke regeneration.^218^ Conceptually, astrocytes (similarly to microglia) express several major phagocytic receptors allowing them to identify dead or dying cells and debris.^219^ Ischaemia arguably upregulates some of these receptors, although astrocytic phagocytosis is tightly coordinated with microglial one; with astrocytes removing small dendrites and microglia engulfing and scavenging soma and main processes.^220^

There is also now a steadily growing interest in understanding and beneficially augmenting the neural plasticity and circuit reorganisation that occurs in areas of spared neural tissue after stroke and that can contribute to substantial recovery of functions.^221,222^ Astrocytes are increasingly recognised as playing important roles in synapse formation, maintenance and turnover in the healthy CNS and also in synapse remodelling after stroke.^44,223–225^ After stroke, astrocytes can undergo variable changes that may result in glucose hypometabolism that reduces the function neighbouring neurones^70^ but may also protect synapses and promote plasticity.^226,227^

### Neuroinfection

#### Bacterial infection

Infectious diseases of the central nervous system are represented by meningitis, encephalitis, myelitis (infection of the spinal cord) and local abscesses. Many different types of pathogens, including bacteria, fungi, protozoa, viruses, and parasites may cause infectious damage to the CNS; however not all of them (or actually only a few of them) can cross CNS defences with ease. Various functional barriers, erected in particular by astrocytes, are highly effective in preventing CNS infection.^228^ When these barriers are compromised, pathogens enter. Some may cross the blood-brain barrier by the paracellular route, by transcytotic mechanisms, inside entering monocytes (the Trojan horse hypothesis), or by other mechanisms, such as hijacking of β-adrenergic receptors, as done for example by *N. meningitides*.^229,230^

The leading response of astrocytes to infectious agents is reactive astrogliosis, which erects parenchymal barriers and encapsulates brain abscesses, thus preventing infection spread. Reactive astrogliosis in neuroinfection is thus defensive and neuroprotective: inhibition of astrocytic reactivity, for example by knocking out GFAP,^231^ or by genetic deletion of NK-1R receptor for substance P, which suppresses reactive astrogliosis that defines resistance to *N. meningitidis* exacerbates the spread of infection and worsens neurological output.^232^ Pathogen-associated molecular patterns (PAMPs) trigger reactive astrogliosis by stimulating pathogen-recognition receptors including several types of Toll-like receptors, TLRs.^233^ Human astrocytes express TLR1 - 5 and TLR9, mouse astrocytes seem to posess all 9 TLRs;^234,235^ expression of TLRs was reported to increase in reactive astrocytes.^236^ Bacteria-derived lipopolysaccharide (which is a canonical PAMP often used to instigate reactive astrogliosis and microgliosis) act as agonists of TLR2 and 4.^237^ Breach of the blood-brain barrier, which accompanies neuroinfection, leads to an extravasation of blood-borne factors which also instigate reactive astrogliosis.^238^ Reactive astrocytes regulate entry and retention of leucocytes, thus controlling inflammatory response. In addition, reactive astrocytes secrete molecules attracting immune cells to the injured region as well as anti- and pro-inflammatory factors regulating neuroinflammation evoked by bacterial invasion.^238^ In focal brain infection, brain abscesses instigate classical inflammatory responses, with reactive astrogliosis, infiltration of macrophages and stromal cells, formation of fibrotic scar and erection of glial perilesional barrier.^239,240^

#### Parasites

Astrocytes are targets for the infection by neurotropic protozoa such as *Toxoplasma gondii* and *Plasmodium falciparum*. Astrocytes infected by *T. gondii* show complex response, including reactive remodelling, secretion of interleukins that reduce parasite burden^241,242^ and start to produce and release kynurenic acid, which is as an endogenous antagonist of NMDA and acetylcholine receptors. Increased production of kynurenic acid may be responsible for an increased risk of schizophrenia in infected patients.^243^ Deletion of astrocyte IL-6 receptor and down-stream JAK-STAT signalling exacerbates the spread of toxoplasma infection and worsens neurological output.^244^ In neuroinfection caused by the malaria parasite *P. falciparum*, astrocytes become damaged which causes a loss of glia limitans, facilitating the spread of infection.^245^

#### Systemic infection and inflammation

Systemic infection associated with septicaemia, is manifested by systemic inflammatory response syndrome, a non-specific complex reaction of the organism to any severe infection, mechanical or thermal injury, or pancreatitis.^246^ Sepsis often triggers a sepsis-associated encephalopathy (SAE) which presents itself with wide symptomatology including reduced attention, disrupted sleep-wakefulness balance, impaired speech and orientation, deficient leaning and memory, numerous perception disorders, focal neurological deficits, seizures, and, in terminal stages, coma.^247^ SAE may develop in two basic scenario: with and without disruption of the blood-brain barrier; in both cases astrocytes, their reactivity and enforcement of the glia limitans are critical.^240,248^ Early stages SAE are often manifested with a ‘sickness behaviour’, an adaptive body response aimed at preservation of energy; signs and symptoms include anorexia, anxiety, irritability, depression, anhedonia, decreased social communication and environmental interest, cognitive changes, including decreased concentration, learning ability and memory.^249,250^

Acute systemic injections of the bacterial antigen lipopolysaccharide (LPS) in mice induces diffuse inflammatory changes in the CNS and mimics this sickness behaviour and is often used as a model to study effects of inflammation on CNS functions. Acute systemic LPS injections induce pronounced but reversible changes in astrocyte gene expression in the prefrontal cortex, along with an approximate 25% decrease in astrocyte branch and process volumes, but with few currently detectable changes in astrocyte physiology and basic preservation of astrocyte core homoeostatic functions such a potassium buffering.^68^ The effects of prolonged LPS exposure on astroglia are less well studied. It deserves mention that LPS is a model for sepsis and should not be extrapolated as a generalised model that is somehow representative of different forms of CNS inflammation. Reactive astrocyte changes induced by LPS differ markedly from those induced by other forms of CNS inflammation such as is associated with autoimmune attack or traumatic injury.^65^

#### Viral infections of the brain

Viral infection targets all cells in the brain, with reactive gliosis dominating the tissue response.^245,251–254^ In addition, astrocytes can be infected and can serve as a viral reservoir. The human immunodeficiency virus (HIV) has significant neurotropism with frequent occurrence of neurological and cognitive symptoms and even HIV-associated dementia.^255^ Although HIV mainly infects and affects microglia, infected astrocytes show decreased homoeostatic capacity.^256^ In contrast, the herpes simplex virus (HSV) preferentially infects astrocytes and oligodendrocytes, whereas microglia become reactive and neuroprotective through release of inflammatory mediators, such as interferon-γ, TNF-α, IL-1, IL-6 and IL-8, RANTES and chemokine CXCL10.^257–260^ Microglia-derived TNF-α, for example inhibits viral replication in astrocytes,^259^ while microglial IL-6 reduces neuronal death. Human cytomegalovirus also affects astrocytes and reduces astrocytic production of thrombospondins,^261^ such affecting synaptogenesis, and suppresses astrocytic expression of glutamate transporters,^262^ thus decreasing neuroprotection again excitotoxic damage. The flaviviruses, such as ZIKA virus and the tick-borne encephalitis virus, selectively infect astrocytes, which become viral reservoirs.^245^ In human astrocytes neurotropic flaviviruses increase autophagy, although viral replication is autophagy-independent.^263^

Clinical manifestations of Coronavirus Disease 2019 (COVID-19), which results from infection with acute respiratory syndrome coronavirus 2 (SARS-CoV-2), include neurological, cognitive and psychiatric manifestations.^264–266^ Pathophysiology of the brain damage caused by SARS-2 includes: (i) direct viral infection of neural cells; (ii) severe systemic inflammation (cytokine storm), with damage to the blood-brain barrier and immune infiltration; (iii) hypoxia associated with respiratory failure; (iv) widespread thrombosis and stroke; and (v) psychological stress linked to disease experience (a kind of post-traumatic stress disorder) and epidemiological interventions.^267^ Both astrocytes and microglia contribute to the pathophysiology of COVID-19.^268^ Astrocytic reactivity was deduced from increased levels of GFAP in the blood plasma of COVID-19 patients^269^ and increased GFAP levels in the white mater in post-mortem tissues of COVID-19 victims with disseminated encephalomyelitis.^270,271^ Post-mortem analysis also revealed clasmatodendrotic astrocytes suggesting that COVID-19 may directly damage astrocytes.^272^ Interrogation of stem-cell derived organoids and organotypic slice cultures revealed preferential infection of astrocytes with SARS-2.^273^ Astrocytes infected with the virus demonstrated signs of reactivity, increased cytokine production and cellular stress. Incidentally these astrocytes did not possess ACE-2, known as a canonical SARS-CoV-2 gate into cells; arguably coronavirus entry factors DPP4 and BSG/CD147 could be involved.^273^

#### Prions

Prion diseases are neurodegenerative conditions represented by Creutzfeldt Jakob’s disease, Gerstmann-Sträussler-Scheinker syndrome, fatal familial insomnia, and Kuru disease.^274^ Prion diseases are caused by accumulation of a pathological prion, known as Prion PrP^Sc^, which are misfolded polypepetides converted form physiologically significant cell-surface glycoprotein PrP^C^ encoded by the *PRNP* gene.^275^ PrP^Sc^ and other prion-like polypepetides have the interesting properties of being infectious and able to induce the generation of more prion particles when seeded from one CNS region to another or from even from one individual to another either by ingestion via the diet, or via implantation into the CNS for example from surgical instruments or tissue grafts.^276^ Pathological prion instigates neuronal death, which in turn causes secondary astrocytic reactivity predominantly in the white matter, but the roles of reactive astroglia in prion diseases are poorly understood and hampered by the emerging complexity of astrocyte responses to prion induced pathologies.^277^

### Autoimmune attack

#### Neuromyelitis optica

Neuromyelitis optica spectrum disorders (NMOSD) are autoimmune diseases that primarily affect and damage myelin and axons in the optic nerve and spinal cord. These disorders include neuromyelitis optica (NMO, also known as unilateral optic neuritis), isolated or recurrent transverse myelitis, longitudinally extensive transverse myelitis or isolated brain lesions with or without detectable anti AQP4-IgG autoantibody. All NMOSD are classified (according to the leading pathophysiological mechanisms) into diseases with identifiable antibodies against aquaporin 4 AQP4-IgG (NMOSD-AQP4), NMOSD without AQP4-IgG or with unknown AQP4-IgG status and NMOSD with identified antibodies against Myelin Oligodendrocyte Glycoprotein, (NMOSD-MOG).^278–280^ About 70% of NMOSD cases are caused by AQP4 auto-antibodies, which after entering the nervous tissue damage astrocytic endfeet rich with AQP4 channels. The remaining 30% of NMOSD are caused by MOG auto-antibodies that target and injure oligodendrocytes. These are neurodegenerative diseases, which besides optic nerve and spinal cord also affect the brain parenchyma and cause neurological and cognitive presentations.

**Fig. 10:**
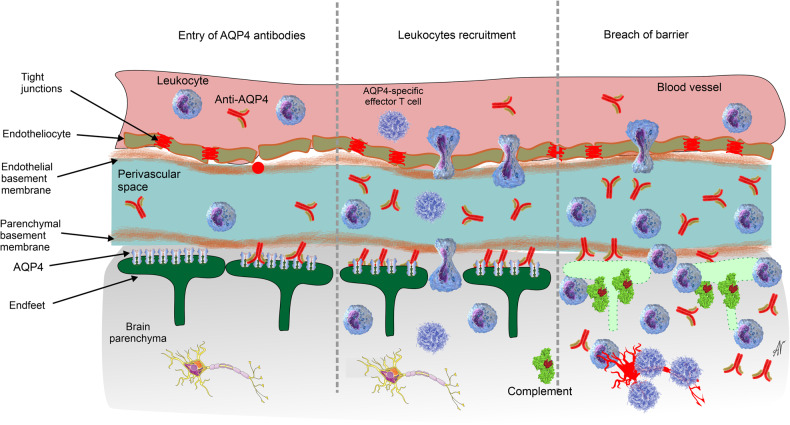
Pathophysiology of the AQP4 form of the neuromyelitis optica. See text for explanation. Modified from ref. ^6^

NMO, also known as Devic’s syndrome (described by Eugene Devic^281^), is the most frequent NMOSD. NMO is mainly caused by auto-anti-AQP4 antibodies^282^ that initially attack astrocytic endfeet,^283^ leading to complement-mediated lysis and destruction of the glial limitans and astrocyte degeneration, which in turn cause the other pathophysiological sequalae (Fig. 10). Thus, NMO can be classified as a primary astrocytopathy. Astrocytic demise is manifested in classical clasmatodendrosis with cell swelling and fragmentation of processes. Massive astrocytic loss precedes demyelination and tissue damage.^284^ Disintegration of glia limitans translates into the widespread damage to the blood–brain barrier and degranulation of natural killer cells that release perforins and granzyme, which in turn, further injure astrocytes and endothelium.^285^ Disruption of the barrier leads to an extravasation of leucocytes and macrophages, reactive microgliosis, neuronal death and full-flown inflammatory response.^85^ In the MOG-associated variant of the disease auto-antibodies attack oligodendrocytes thus causing their death and secondary astro- and microgliotic response.^286^ Incidentally, the anti-AQP antibodies also attack kidneys, and patients with AQP4 auto-antibodies form of NMOSD demonstrate lover glomerular filtration rate.^287^

#### Multiple sclerosis

Multiple sclerosis (MS) is a chronic autoimmune inflammatory disease with antibodies primarily attacking myelin sheaths and causing multiple demyelinated areas in white and grey matter throughout the CNS (with most frequent localisation in the optic nerve, spinal cord, brain stem, periventricular white matter, and the grey matter near the subarachnoid space); these lesions are considered to be histopathological hallmarks of the disease.^288–290^ Immune attack on the nervous tissue begins from the entry of auto-reactive T lymphocytes and accumulation of auto-antibodies. With breach of the blood-brain barrier, immune cells infiltration as well as extravasation of blood-borne molecules such as fibronectin triggers, together with the death of oligodendrocytes, degeneration and transection of axons and starts a multicellular inflammatory response associated with reactive astro- and microgliosis.^291–294^ Focal inflammation evolves into fibrotic scars surrounded by glial borders and barriers; which are the substrate of mature sclerotic lesions. Interrogation of specific cell-cell interactions using new tools such as bar-coded viral tracing technology, is beginning to identify specific molecular interactions, for example that Sema4d and EphrinB3 expressed by microglia control astrocyte responses respectively through PlexinB2 and EphB3 receptors.^295^

Astrocytes play central and heterogeneous roles in the regulation of CNS inflammation in autoimmune diseases such as MS and its experimental models.^152,296^ Astrocytes in MS undergo both reactive and atrophic changes. Reactive astrogliosis is a prominent feature of MS lesions with reactive astrocytes surrounding active demyelinating foci.^291,297^ In the animal model of MS, experimental autoimmune encephalomyelitis (EAE), astrocytes are found around demyelinating areas but may even occur before emergence of the latter.^298,299^ Reactive astrocytes secrete various cytokines and are involved in recruitment of leucocytes traffic into the brain parenchyma.^300,301^ Recent findings suggest that exposure to certain environmental factors such as pesticides can augment astrocyte proinflammatory signalling and thereby promote CNS autoimmune inflammation.^302^ Notably, microbial metabolites produced by commensal gut flora can limit pathogenic activities of microglia and astrocytes and suppress CNS inflammation in MS experimental models^303^ and can contribute to the induction of specific LAMP1+TRAIL+ astrocytes that limit CNS inflammation by inducing T cell apoptosis.^304^

Fibrotic scar is a common component of MS mature lesions; active lesions are enriched in collagen-producing cells, aggregates of fibronectin and collagen, mesenchymal perivascular aggregates of platelet-derived growth factor receptor (PDGFR)β-bearing cells, and pro-fibrotic factors such as biglycan and decorin.^305,306^ In contrast to TBI, the MS scar is mainly produced by resident fibroblasts and not by vascular infiltrating stromal cells.^89^ Development of MS is also associated with direct damage to astrocytes. Astrocytes with swollen somata and processes surround blood cells infiltration, with some of them showing signs of clasmatodendrosis.^297,307^ In addition, astrocytes lose AQP4 channels in their endfeet and down-regulate expression of glutamate transporters, thus adding to excitotoxic damage of oligodendrocytes.^308^ Experimentally-induced depletion or attenuation of reactive astroglia markedly exacerbates the clinical progression, spread of inflammation and tissue loss in EAE during the induction phases of the disorder,^81,309,310^ whereas similarly induced depletion of proliferating reactive astrocytes at later times during the progressive phase ameliorates disease, and this amelioration is at least in part due to removal of astrocyte Ccl2 production.^310^ Similar effects are observed after transgenic deletion of Ccl2 specifically from astrocytes, which showed little effects early in EAE but demonstrated that astrocyte Ccl2 sustains disease symptoms and inflammation during chronic EAE, making it a therapeutic target.^311^ Such findings point towards different roles and different effects of reactive astrocytes at different times during the progression of autoimmune inflammation and suggest that different therapeutic approaches involving astrocytes may be required at different times. These observations also highlight that therapeutic approaches should be directed at specific aspects of astrocyte reactivity, for example astrocyte Ccl2 production, rather than at attenuating reactivity per se, which may lead to unexpected and undesirable consequences. New technologies are allowing selective identification of subsets or sub-states of reactive astrocytes, and have identified a role for a potentially therapeutically targetable mechanism that limits XBP1-driven pathogenic astrocyte responses.^312^

### Conclusions

Injury or damage to CNS tissue of a mechanical, vascular, infectious or auto-immune nature triggers diverse inflammatory responses. Reactive astrogliosis plays essential roles in both attracting and containing neuroinflammation, and is critical for wound closure, fibrotic scar formation, erecting glia limitans barrier delineating the damaged tissue and supporting postlesional regeneration and plasticity.

## Genetic astrocytopathies

### Alexander disease

Alexander disease, or AxD (named after its discoverer W. Steward Alexander^313^) is an incurable genetic astrocytopathy caused by sporadic mutations in the GFAP gene.^124^ Astrocytic expression of mutant GFAP proteins results in a profound loss of white matter, i.e. AxD is a genetic leukomalacia. Histological hallmarks of AxD are the emergence of perivascular Rosenthal fibres consisting of ubiquinated aggregates of GFAP, vimentin, small heat shock proteins αβ-crystallin and Hsp27, and plectin.^124,314^ How expression of mutated GFAP in astrocytes translates into severe white matter deficiency remains unknown.^315^ Astrocytes in AxD show profound alterations in morphology and function, with evidence of abnormal glutamate clearance, substantial cell stress in the form of upregulated expression of heat shock protein, MAPK pathways, JNK and p38 kinases, as well as increased autophagy and proteasomal activity.^124,316^

### Duchenne muscular dystrophy

Duchenne muscular dystrophy (named after Guillaume-Benjamin-Amand Duchenne de Boulogne who provided early and comprehensive description of clinical presentation and histopathology of this disorder^317^) is an X-linked recessive neuromuscular disorder caused by mutations in the *DMD* gene that encodes the protein dystrophin. Dystrophin is a main part of the dystrophin-associated protein complex (also known as a costamere) essential for contraction of the striated muscle.^318,319^ Expression of mutant *DMD* gene results in progressive muscle degeneration leading to various problems with locomotion, ambulation, as well as deficits of cardio-vascular and respiratory systems.^320^ In addition to muscular manifestations, Duchenne muscular dystrophy is often associated with psychosocial abnormalities and cognitive impairment, while histopathologically neuronal death and dendritic abnormalities are frequently observed post-mortem.^126^ In the CNS, dystrophin is mainly expressed in protoplasmic astrocytes in neocortex and in velate astrocytes and Bergmann glia in the cerebellum; with particularly high expression in perivascular astrocytic endfeet.^321,322^ At the cellular level dystrophin scaffolding network supports morphologically complex astrocytic processes and defines correct localisation, clustering and density of numerous channels, receptors and transporters. In particular, dystrophin-associated protein complex links AQP4 aquaporin channels and K_ir_4.1 channels in the endfeet: one of the key components of astrocytic homoeostatic hardware.^323,324^ Astrocytes differentiated from pluripotent stem cells isolated from Duchenne muscular dystrophy patients (and carrying mutant *DMD* gene) were characterised by abnormal cytoskeleton, severely deficient glutamate uptake, and compromised K^+^ buffering.^127^ Thus, the Duchenne muscular dystrophy is a primary genetic astrocytopathy that impairs synaptic transmission, causes excitotoxicity and secondary neurodegeneration.

### Conclusions

The whole range of primary genetic astrocytopathies is yet to be fully characterised. Mutations in genes encoding astrocyte-specific proteins can lead either to loss of functions with subsequent secondary neural injury or to the emergence of aberrant cellular phenotypes damaging nervous tissue in yet unidentified manner.

## Epilepsy and migraine

### Epilepsy

Epilepsy manifested by seizures originates from an uncontrolled over-excitation of motor brain areas.^325^ At the cellular level this overexcitation stems from a slow synchronous depolarisation of neurones, known as paroxysmal depolarisation shift (PDS) within the epileptic foci. The PDS develops from large and relatively slow excitatory postsynaptic potentials mediated by AMPA and NMDA glutamate receptors activated by aberrant and large glutamate release within the foci.^326^ Astrocytes regulate glutamate presence in the interstitium,^327^ tune neuronal excitability through K^+^ buffering,^328^ and influence inhibitory/excitatory balance through tonic release of GABA^329^ and by supplying Cl^-^ to inhibitory synapses.^330^ Astrocytes are severely affected in epileptic foci and abnormal astrocytic homoeostasis contributes to ictogenesis.^331,332^ In mesial temporal lobe epilepsy or tuberous sclerosis, astrocytes demonstrate a specific form of reactivity characterised by an increased expression of GFAP^333^ and morphological atrophy manifested by reduced complexity of arbour and loss of distal leaflets.^334^ These morphological aberrations are paralleled with loss of function. Loss-of-function missense mutations as well as single nucleotide polymorphisms in the genes encoding K_ir_4.1 and AQP4 (which is selectively astroglial and operates in concert with K^ir^4.1) are linked to epilepsy in humans.^335^ Astrocytes in post-mortem samples of patients with mesial temporal lobe epilepsy show significant down-regulation of K_ir_4.1 channels critical for K^+^ buffering.^335^ In experimental settings, conditional knockout of K_ir_4.1 results in epileptic phenotype.^336,337^ Failure of glutamate homoeostasis in epilepsy reflects significant down-regulation of astrocytic glutamate transporters and glutamine synthetase, the two principal components of the glutamate (GABA)-glutamine shuttle. In temporal lobe epilepsy, levels of astrocytic glutamate transporters in hippocampus are reduced by up to 40%.^338^ Similar decreases in astrocytic expression of glutamate transports are characteristic for animal models of epilepsy,^339,340^ and genetic ablation of EAAT1/2 triggers seizures.^338,341^ Astrocytic glutamine synthetase is reduced in post-mortem tissues from epilepsy patients.^342^ Arguably, decreased glutamine supply limits GABA release, thus increasing neuronal excitability.^343^ Reduced astrocytic homoeostatic support is also evidenced by the loss of endfeet polarisation of AQP4^344^ and decreased expression of monocarboxylate transporter 1^345^ responsive for lactate supply of neurones. Increased APOE expression and excessive lipid accumulation in astrocytes promote neuronal hyperexcitability and disease progression in temporal lobe epilepsy.^346^ Another astrocyte-specific mechanism involved in ictogenesis is linked to gap junction forming connexion Cx43 channels, which are down-regulated in epilepsy.^347,348^ Uncoupling of astrocytic syncytia or knocking out Cx43 in experimental models instigates seizures.^348,349^ It is also noteworthy that CTE can lead to atypical astrocyte responses that contribute to recurrent seizures.^185^

### Familial hemiplegic migraine

Familial hemiplegic migraine FHM type 2, clinically manifested as migraine with aura, is linked to loss-of function mutations of the*ATP1A2* gene encoding the astrocyte-specific α2 subunit of Na^+^/K^+^ ATPase (NKA). Astrocytic NKA is central for K^+^ buffering^350,351^ and for astrocytic Na^+^ signalling.^352^ In the context of FHM type 2 deficient NKA not only results in the impaired K^+^ buffering but is also linked to down-regulation of astrocytic EAAT2 glutamate transporters, which dives pathophysiology of this form of migraine.^353^

### Conclusions

Loss of astrocytic homoeostatic support is the primary mechanism underlying neuronal hyperexcitability in epilepsy and in migraine. In the case of epilepsy, impaired K^+^ buffering, glutamate clearance, lipid accumulation and malfunctioning of the glutamate (GABA)-glutamine shuttle emerge as leading pathophysiological processes. Familial hemiplegic migraine type 2 is linked to the loss of function mutations of astrocytic Na^+^/K^+^ ATPase which leads to an abnormal regulation of interstitial K^+^ and glutamate.

## Astrocytes as main target of toxic encephalopathies

### Hyperammonaemia and hepatic encephalopathy

Hepatic encephalopathy is a primary astrocytopathy caused by an increased level of blood ammonium. This increase is observed in several diseases including congenital deficits in urea cycle enzymes, Reyes syndrome in children, uraemic encephalopathy, diabetic encephalopathy, or hypoglycaemic encephalopathy, although the most frequent cause for hyperammonaemia is associated with acute (due to poisons or drugs) and chronic (cirrhosis) liver failure.^354^ Ammonium is mainly produced in the gut by amino acid deamination with subsequent conversion to ammonium by urease containing bacteria and is detoxified in the liver by the urea cycle enzymes and by glutamine synthetase, highly expressed in hepatocytes.^355^ Physiological levels of ammonium in the blood are about 10–20 μM, but following liver failure ammonium rise to millimolar levels.^356,357^ Ammonium crosses the blood-brain barrier with ease (in gaseous NH_3_ form), and in this manner liver failure causes its massive increase in the brain. Hyperammonaemia triggers numerous neurological and neuropsychiatric symptoms, including poor concentration, impaired memory and cognition, psychotic presentations, sleep disturbance and lethargy, and stupor and coma in severe cases.^358^ In its terminal phase, hyperammonaemia leads to brain oedema which may even cause brain herination, and this progressive oedema is the main case of death.

Astrocytes are the exclusive possessors of glutamine synthetase in the brain,^137^ which makes them the main target for ammonium. Most of ammonium entering the brain is converted to glutamine, which causes glutaminosis^359^ and effectively lowers brain parenchymal ammonium concentrations, but which also maintains the concentration gradient and favours additional entry of ammonium from the blood to the brain.^360^ At the cellular level, excess of ammonium mainly affects astrocytes with neuronal injury being secondary, resulting mainly from failure of astrocytic homoeostatic cascades and oedema. The histopathological hallmark of ammonium toxicity is manifested by the emergence of aberrant astrocytes, known as Alzheimer type II astrocytes. These pathological astrocytes were initially described by Carl Von Hösslin and Alois Alzheimer^361^ during post-mortem analysis of the brain of a patient with toxic copper encephalopathy, known today as a Wilson’s disease. The term Alzheimer astrocytes type II was introduced in 1942.^362^ Alzheimer’s type II astrocytes are characterised by (i) increased size of somata, primary processes and swollen endfeet; (ii) increased nuclei; (iii) decreased electron density of the cytoplasmic matrix in perikaryon, processes, and endfeet; (iv) increased density of mitochondria and enlarged endoplasmic reticulum in soma and primary processes; (v) less compact bundles of intermediate filaments.^357,363^ In addition, there are mild signs for astrocytic reactivity mainly in cerebral neocortices.^364^ The affected astrocytes demonstrate profound loss of function, in particular impaired K^+^ buffering,^365,366^ compromised H^+^ transport,^367^ and substantial decrease in glutamate uptake.^368^ Astrocytes exposed to excessive ammonium start to generate aberrant Ca^2+^ signals, which may cause excitotoxic release of glutamate.^369,370^

Increased activity of glutamine synthetase and increased production of glutamine are key pathophysiological features of ammonium neurotoxicity. Excessive glutamine is accumulated in astrocytes and converted back to glutamate and ammonium by mitochondrial phosphate-activated glutaminase. Overload of mitochondria with ammonium instigates over-production of reactive oxygen species and opening of mitochondrial permeability transition pore, which further damages astrocytes and induce astrocyte swelling; this sequence of events is known as the ‘Trojan horse’ hypothesis of hepatic encephalopathy.^371^ High ammonium also down-regulates astrocytic expression of glutamine exporting SNAT3/SLC38a3 transporter thus exacerbating glutamine retention.^372^ All these astrocytic changes result in considerable and often irreversible neuronal damage, with about 50% of patients demonstrating persistent neurological manifestations even after liver replacement and normalisation of blood ammonium.^355^

### Trace metals toxic encephalopathies

Contact with many metals, such as arsenic (As), manganese (Mn), mercury (Hg), lead (Pb), aluminium (Al), nickel (Ni), bismuth (Bi), cadmium (Cd), zinc (Zn), copper (Cu) and iron (Fe), may evoke acute or chronic toxic encephalopathies; in addition, chronic accumulation of such metals is known to increase the risk for neurodegenerative disorders, particularly of AD and PD.^105^ Neurological symptoms of metal encephalopathies are quite variable and may include sleep abnormalities, disorientation, visual abnormalities, sensory lesions, cerebellar ataxia, hearing loss, weakness, tremor, memory problems and cognitive decline. In the brain, the excess of heavy metal is almost completely removed by astrocytes^373^; accumulation of metals in astrocytes impairs their homoeostatic capabilities, which in turn, damages neurones and results in neurological symptoms.

Iron is probably one of the most important metals sustaining critical physiological processes, but also with the potential to cause neurotoxicity when in excess. In the CNS, iron is accumulated mainly by astrocytes and to a somewhat lesser extent by microglia. This accumulation is mediated by plasmalemmal transporters predominantly expressed in glial cell, such as divalent metal transporter 1 DMT1/SLC11A2 or zinc transporter ZIP14/SLC39A14,^374,375^ which both transport Fe^2+^ (ferrous), whereas Fe^3+^ (ferric) is accumulated through transferrin receptors.^375^ In physiological contexts, astrocytes provide iron storage in the form of ferritin, and when needed, iron is released by ferroportin and ceruloplasmin ferroxidase.^376^ Ceruloplasmin deficiency results in brain iron overload and neurotoxicity.^377^

Iron overload, following increased intake, which may in particular result from the use of iron-containing implants widely employed in orthopaedic surgery,^378^ or can occur after haemorrhagic stroke, results in compensatory upregulation of glial iron transporters, and down-regulation of neuronal iron transporters, both aimed at neuroprotection.^378^ Astrocytic overload with iron however significantly reduces glutamate clearance by down-regulation expression of EAAT1 glutamate transporters^379^ and impairs operation of the glymphatic system.^380^

Another primary astrocytopathy linked to dysregulated iron homoeostasis is aceruloplasminemia, an autosomal recessive neurodegenerative disease caused by a loss of function mutations of the gene encoding ceruloplasmin, an enzyme that converts ferrous iron to ferric. Neurological presentations of aceruloplasminemia include ataxia, involuntary movement, cognitive dysfunctions and parkinsonism.^381^ In the CNS, ceruloplasmin is expressed almost exclusively in astrocytes, and its deficiency results in iron overload and formation of iron deposits. Oversized and deformed astrocytes are the histopathological hallmark of the disease.^382^ Impaired astrocytic iron homoeostasis triggers secondary neuronal iron toxicity, which together with loss of astrocytic homoeostatic support, triggers neuronal death.

Mercury causes severe brain abnormalities, for example in Minamata disease.^383^ Molecular pathophysiology of mercury toxicity is defined by an induction of reactive oxygen species production and down-regulation of anti-oxidant defence. Astrocytes are the main depository for mercury,^384^ which they accumulate by large neutral amino acid transporter LAT1/SLC7A5.^385^ Astrocytic overload with mercury causes cell swelling, it severally impairs glutamate uptake, K^+^ buffering and glutathione production,^386^ this causing secondary excitotoxicity and oxidative damage to neurones. The neurotoxicity of other metals, such as lead, aluminium or manganese are similarly mediated through astrocytic loss of function, as all these metals are primarily accumulated into astrocytes. In particular, exposure to lead results in a significant down-regulation of astrocytic glutamate transporters^387^ and in cell swelling possibly because of increased water transport through AQP4 channels.^388^ Similarly, aluminium and manganese reduce astrocytic glutamate uptake, thus leading to excitotoxicity and secondary neuronal death.^389–391^

### Environmental toxins and neurodegenerative disorders

Exposure to various types of environmental toxins including trace metals as just discussed, but also pesticides such as organophosphates, substances of abuse such as methamphetamine, organic solvents, air pollutants, and others are increasingly recognised as risk factors in neurodegenerative disorders such as PD and others.^392,393^ Many of these substances induce widespread changes in astrocyte functions, including loss of functions and dysregulated astrogliosis, which in turn may serve as comorbidities in degenerative conditions such as AD, PD, and others.^105,393–395^

### Conclusions

In summary, toxic encephalopathies are primary astrocytopathies, in which toxic substances are accumulated by and damage astrocytes, with neuronal loss being secondary to astroglial homoeostatic failure. Given the essential role of astrocytes in clearing and detoxifying many substances and the changes in astrocyte functions that such substances can evoke, it is interesting to consider (and worthy of more extensive investigation) that chronic exposure to trace amounts of such substances might act as risk factors and comorbidities in a variety of CNS neurodegenerative conditions.

## Neuropsychiatric disorders: predominance of astrocytic atrophy and loss of function

### Mood disorders

#### Major depressive disorder (MDD)

Decreases in the total number of glial cells and astrocytes are the main and most consistent histopathological hallmark of MDD post-mortem brains. Such decreases have been documented by both stereological and immunocytochemical studies. Decreases in astrocyte densities were observed in many brain regions including hippocampus, amygdala, prefrontal and anterior cingulate cortex.^106,108,396–399^ Astrocytes in post-mortem brains and in animal models of depression show atrophic morphology. Vimentin-positive astrocytes in the prefrontal white matter show fewer primary processes^400^; astrocytes in the grey matter lose ~ 50% of their perivascular endfeet.^401^ In addition, MDD is associated with significant decreases in astrocytic glutamate transporters and glutamine synthetase, impairing neurotransmission.^402,403^

**Fig. 11:**
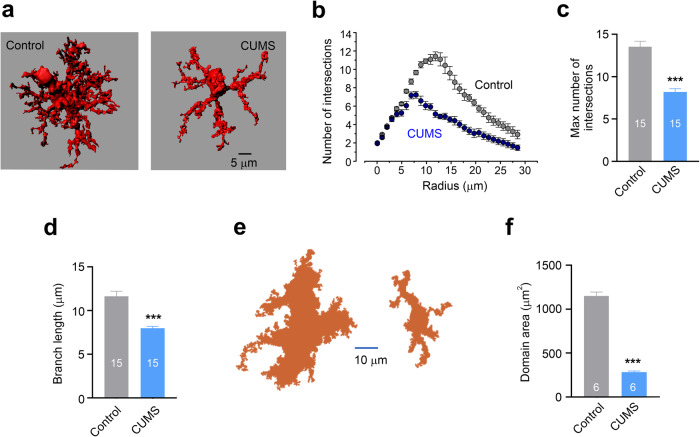
Exposure to Chronic Unpredictable Stress (CUMS) induces morphological atrophy in prefrontal cortex astrocytes in mice. **a** Representative 3D reconstruction of astrocyte in control and CUMS groups. **b** Sholl analysis of astrocytic morphology for control and CUMS groups shows the number of intersections of astrocytic branches with concentric spheres centred in the middle of cell soma. **c** Maximal number of intersections for astrocytes in control and CUMS groups. **d** Average length of astrocytic processes in control and CUMS groups. **b**–**d** *n* = 15 for each group. **e** Representative examples of astrocytic territorial domains obtained as a projection of astrocytes along the z-axis projection for control and CUMS animals. **f** Average astrocytic domain area (E) and average length of astrocytic processes for control and CUMS group. All data are presented as mean ± s.e.m. *p < 0.05, **p < 0.01, ***p < 0.001. The number of experiments is indicated on each column. Reproduced from ref. ^409^

In experimental models of depression, which are mainly based on exposure to unpredictable or social stress, numbers of GFAP-positive astrocytes, as well as GFAP levels in cortex and hippocampus, are significantly reduced.^404,405^ These morphological changes are accompanied by decreased astrocytic metabolism and glutamate uptake.^406,407^ Detailed analysis of astrocytic morphology using genetic reports (either ALDH1L1-eGFP reporter mice or viral transfection with astrocyte-targeted mCherry) revealed significant decrease of astrocytic complexity and shrinkage of astrocytic territorial domains in prefrontal cortex (Fig. 11,^408,409^). This structural atrophy was associated with a decrease in astrocytic expression of linker ezrin^409^; ezrin is essential for astrocytic morphological plasticity and extension of astrocytic leaflets.^410,411^ Anti-depressant treatment with fluoxetine or with specific acupuncture alleviated depressive behaviours, rescued astrocytic atrophy and restored ezrin expression.^409^ In Flinders Sensitive Line rats, which spontaneously develop depression-like phenotype, hippocampal GFAP-positive astrocytes are smaller and have atrophic arborisation.^412^ Likewise, astrocytes with small cell bodies and less complex processes were identified in Rhesus macaques with a self-injurious behaviour.^413^

Partial ablation of astrocytes in the prefrontal cortex of healthy rats (by injection of gliotoxin L-α-aminoadipic acid) resulted in the development of depressive-like behaviours, whereas injection of neurotoxin ibotenate did not have such an effect.^404^ Ablation of astrocytes also caused secondary damage to neurones translated into impaired working memory and learning.^414^ Very similar effects were caused by inhibiting glial glutamate transporters in the prefrontal cortex.^415^ At the same time, boosting astrocytic glutamate uptake with riluzole^406^ or ceftriaxone restored depressive behaviours triggered by chronic stress.^416^ Experimental models of depression also demonstrate uncoupling of astrocytic syncytia, whereas pharmacological inhibition of gap junctions by intracerebral infusion of carbenoxolone induced anhedonia and anxiety-like behaviour.^417^ Chronic stress additionally down-regulates astrocytic expression of AQP4.^418^

#### Bipolar disorder (BP)

Both the total number of glial cells and astrocytic densities are substantially (up to 40%) decreased in the post-mortem BP tissues, whereas numbers of neurones are not changed.^419–422^ Loss of astrocytes leads to an overall decrease in neuroprotection and affects neurotransmission.^423^ Astrocytic atrophy and reduced synaptic coverage might be responsible for hyperactive glutamatergic transmission often observed in BP, especially during mania phases.^424–427^

#### Post-traumatic stress disorder (PTSD)

A substantial (and increasing) number of people experience PTSD after various forms of traumatic experience. Pathological changes in astrocytes in various models of PTSD are similar to those in models of depression.^110^ For example, GFAP levels as well as densities of GFAP-positive astrocytic profiles are decreased in a single prolonged stress model of PTSD.^428^ Astrocytes in experimental PTSD model show significant atrophy with decreased processes complexity and the appearance of fusiform morphotypes.^429^

#### Anxiety disorders

In anxiety, astrocytes display generalised atrophy similar to that seen in other mood disorders. For example, about 60% of astrocytic perivascular endfeet are lost in the prefrontal cortex in high anxiety-like behaviour rats developing endogenous anxiety.^430^ Astrocytes in these rats have fewer processes and less complex arborisation.^430,431^ Anxiety may also be linked to astrocytic glutamate uptake deficiency, as intra-amygdala injections of glutamate transporter inhibitor dihydro-kainic acid causes anxiety behaviour.^415^

#### Antidepressants target astrocytes

Antidepressant drugs include (i) selective serotonin reuptake inhibitors (SSRI), of which fluoxetine (Prozac) is the most popular, (ii) serotonin/noradrenaline reuptake inhibitors (SNRI), (iii) tricyclic antidepressants, (iv) inhibitors of monoaminoxidase, (v) lithium, and (vi) ketamine. Lithium, valproic acid (VPA), and carbamazepine (CBZ) are used as anti-bipolar drugs. The SSRI, which inhibit serotonin transporter SERT/SLC6A4 (expressed in both neurones and astroglia) and thus increase bioavailability of serotonin, are probably the most widely used in the treatment of depression. Besides acting on neurones, fluoxetine and other SSRIs affect astrocytes; in the context of major depression, SSRI treatment reverses both astrocytic atrophy and the decrease in astrocyte numbers in animal models.^405,432^ Incidentally, acupuncture at the Zusanli (ST36) acupoint that is used to treat depressive symptoms, prevented the development of both astrocytic atrophy and depressive-like behaviours in mice subjected to chronic stress regimen.^409^ Surprisingly, the main molecular target of SSRIs in astrocytes is not the SERT but rather 5-HT_2B_ serotonin receptors. Treatment with fluoxetine upregulates expression of 5-HT_2B_ receptors (which is decreased by chronic stress) selectively in astrocytes, and not in neurones.^433^ More importantly however, fluoxetine acts as a powerful and specific agonist of astrocytic 5-HT_2B_ receptors. Activation of these receptors by fluoxetine triggers several signalling cascades.^434–436^ In addition, fluoxetin-induced activation of astrocytic 5-HT_2B_ receptors induces transactivation of epidermal growth factor receptors, which, in turn, recruit MAPK/ERK or PI3K/AKT signalling cascades to regulate expression of several genes (such as Ca^2+^-dependent phospholipase A2, cPLA2, subtype 2 of adenosine deaminases acting on RNA’s, ADAR2, or subtype 2 of kainate receptors, GluK2) related to mood disorders.^437,438^ Fluoxetine also normalises interstitial pH, which is affected in mood disorders, by phosphorylating and stimulating astrocytic Na^+^-H^+^ transporter NHE/SLC9a1.^439^

Lithium, which is an effective treatment against acute mania and depression in the context of BP, has several cellular targets. In particular, lithium inhibits glycogen synthase kinase 3β,^440^ which translates in an increase of the density and complexity of astrocytes in the animal models of depression.^441^ Lithium also regulates astrocytic morphology through the extracellular matrix regulatory enzyme lysyl oxidase (LOX) and peroxisome proliferator-activated receptor γ, with *LOX* being the most highly regulated lithium-responsive astroglial gene.^441^

The central anaesthetic ketamine is an inhibitor of NMDA receptors, with surprisingly potent and rapid anti-depressant action.^442^ Research into ketamine actions in the CNS revealed multiple effects, including potentiation of synaptogenesis, increased density of dendritic spines and upregulation of postsynaptic levels of AMPA receptors.^443^ In a rodent model of depression, treatment with ketamine alleviated aberrant behaviours and restored astrocytic atrophy.^412^ Ketamine action on the morphology of astrocytes is mediated through cAMP intracellular signalling: ketamine directly increases cytoplasmic cAMP without activation of metabotropic receptors.^444^ Ketamine also potentiates cholesterol transport from astrocytes to neurones, thus stimulating synaptogenesis and morphological plasticity.^444^

### Obsessive compulsive disorders (OCD)

Obsessive compulsive disorders (OCD), such as for example Tourette syndrome or grooming disorders, are clinically presented by repetitive behaviours or compulsions and intrusive thoughts, or obsessions.^445^ The pathophysiology of OCD revolves around aberrant excitatory-inhibitory balance.^446^ Astrocytes are fundamental for both excitatory and inhibitory transmission through glutamate glutamate (GABA)-glutamine shuttle^447^ and chloride ionostasis ^330^ and are thus well positioned to contribute to OCD pathophysiology.^448^ Indeed, deletion of EAAT1 astrocytic glutamate transporter translates into OCD-like phenotype manifested by excessive self-grooming and repetitive tic-like head shake^449^ arguably linked to hyperactive resting state activity and aberrant functional connectivity of the cortico-striatal-thalamic circuitry as revealed by diffusion functional magnetic resonance imaging.^450^ Experimentally reducing astrocyte calcium signalling in striatal circuits induces OCD-like repetitive behaviours in rodents.^451^ Aberrant astrocytic glutamate homoeostasis may also be linked to Tourette syndrome.^452^ Recently the role of astrocytic protein SAPAP3, encoded by Dlgap3 gene, in pathophysiology of OCD was suggested based on in depth analysis of astrocytic proteome. The SAPAP3 regulates astrocytic morphology, their territorial domains and notably the volume and extension of perisynaptic leaflets.^453^

### Schizophrenia

Astrocytic changes in schizophrenia are rather mild. There are no signs of reactivity, instead there are observations of a generalised decrease in the density of astrocytes in cortical and subcortical areas, and even more prominent astrocytic depletion in the white matter.^454–456^ Dystrophic and swollen astrocytes were detected in electron microscopy of post-mortem brains of schizophrenic patients.^457^ Similarly post-mortem analysis revealed decreased levels of astrocytic EAAT1/2 glutamate transporters.^458,459^ Animals with genetic deletion of astrocytic glutamate transporters show some schizophrenia-like behaviours.^460^ Astrocytes may contribute to the pathophysiology of schizophrenia through an increased production of NMDA receptor blocker kynurenic acid, which may also link risk of schizophrenia with infection as described in the previous chapter. Finally, there is evidence for an abnormal and delayed maturation of astrocytes derived from stem cells obtained from schizophrenia patients. These astrocytes show less complex morphology and functional asthenia^461^ and may be a part of generalised glial deficiency in embryonic development that is linked to schizophrenia.^462^

### Addictive disorders

Glutamatergic transmission regulates reward circuitry and dopaminergic connectivity. Glutamatergic transmission is controlled and regulated by astrocytic glutamate-glutamine shuttle, thereby providing a means by which astrocytes may contribute to the pathophysiology of addictive disorders through glutamatergic pathways.^463^ Experimental models of addictive disorders demonstrate significant morphological atrophy of astrocytes in nucleus accumbens.^109,464^ Both GFAP-positive astrocytic profiles and 3D reconstructions of astrocytes expressing green florescent protein in the nucleus accumbens of rats addicted to cocaine show atrophic morphology, with the volume of reconstructed cells decreasing by 19% and surface area by 21% as well as reduced synaptic coverage.^107^ Reduction of the association of astrocytic leaflets with synapses is linked to a decreased expression of ezrin (which is responsible for leaflets extension), while deletion of ezrin increased addictive behaviour.^464^ In addition to morphological dystrophy, development of addiction is associated with significant decrease in expression of astrocytic glutamate transporters, reduced glutamate clearance, increased levels of glutamate and increased glutamate spillover in nucleus accumbens.^465–467^ Moreover, addiction build-up seems to be linked to astrocytic NMDA receptors, such that deletion of the NR2C subunit specifically from astrocytes potentiated extinction of cocaine preference memory.^468^ Generalised astrocytic atrophy is also characteristic for alcoholism, in which astrocytes display loss of function, such as for example reduction in synthesis and secretion of thrombospondins.^469–472^

### Conclusions

Neuropsychiatric diseases are characterised by widespread astroglial asthenia, with decreased cell numbers, morphological atrophy, and loss of function. Ablation of astrocytes, or inhibition of astrocyte specific homoeostatic molecules in experimental animals trigger behavioural abnormalities that resemble neuropsychiatric symptomatology in humans. Despite widely popularised views of the inflammatory nature of psychiatric diseases, there is little evidence corroborating neuroinflammatory changes of CNS tissue.

## Neurodevelopmental disorders

### Down syndrome

Down syndrome (named so after John Langdon Down who described this syndrome under the name of ‘Mongolism’ in 1866^473^) is the genetic disorder caused by an extra copy of chromosome 21. Clinically Down syndrome is manifested by hypothyroidism, malformation of the cardiac system and gastrointestinal tract, abnormal hearing and vision, and aberrant development of the brain resulting in severe intellectual disability.^474^ The neocortex of Down syndrome individuals is characterised by profound (30–50%) decrease in the number of neurones and neuroglia.^475^ Aberrant and premature developmental gliogenesis (which ultimately affects both neurones and glia) lies at the core of the cellular pathophysiology of Down syndrome.^476,477^ This abnormal gliogenesis is linked to a deficit in Sonic hedgehog (Shh) signalling. Even a single injection of an Shh agonist was shown to rescue neuronal numbers and behavioural deficits in an animal model of the syndrome.^478^ The gene encoding astrocyte-specific protein S100B is located in chromosome 21 and increased levels of S100B are characteristic for Down syndrome.^479^ Another cascade linking astrocytes to the pathophysiology of Down syndrome is an increased production of hydrogen sulphide, which is catalysed by cystathionine-β-synthase expressed, more or less exclusively, in astrocytes, and upregulated in a rat model of the disease. Inhibition of this enzyme was claimed to rescue pathological behaviours.^480^

### Spina bifida

Spina bifida (the split spine in Latin) arises from pathological embryogenesis, in which the neural tube fails to close. Clinical manifestations are multifaceted and include disabilities with motor, urinary, intestinal, sexual and mental presentations.^481^ Neurodegeneration and abnormal nervous tissue development is, arguably, triggered by the invasion of amniotic fluid into the developing spinal cord and adding to the mechanical trauma. In particular, spina bifida is characterised by premature astrogliogenesis, which affects neuronal development and instigates reactive astrogliosis.^482^

### Astrocytic RASopathy

Mutations of rat sarcoma virus or RAS proteins are behind a class of disorders known as RASopathies.^483^ Frequently, RASopathies present cognitive deficits and delayed cognitive development. The pathophysiology of RASopathies is centred on excessive foetal astrogliogenesis and prevalence of functionally deranged astrocytes.^484,485^ In Costello syndrome, which is a form of RASopathy caused by mutations of the gene encoding HRAS protein, astrocytes are hypertrophic and demonstrate increased proliferative activity; it was argued that these astrocytes increase synaptogenesis through elevated secretion of synaptogenic factors and premature formation of perineuronal net.^486^

### Intellectual disability

Intellectual disability (ID) is a heterogeneous genetic disorder in children with an incidence of 1–3%, representing a substantial public health burden.^487,488^ Familial studies have revealed a relatively large number of X-linked ID (XLID) forms which may explain the excess of affected males.^489^ In families affected by XLID, a number of loss-of-function mutations were found in the *GDI1* gene,^490^ which encodes a cytosolic protein αGDI, the RAB GDP dissociation inhibitor, involved in the control of the guanosine diphosphate (GDP)-bound form of the brain RAB GTPases^491,492^ and regulates vesicle dynamics.^493,494^ Two isoforms of *GDI* are present: *GDI1* encodes for αGDI, principally enriched in the brain, whilst *GDI2* encodes for βGDI that is ubiquitously expressed.^495^

Initial studies of the role of *GDI1* were focused on neurones. Neuronal migration and differentiation were impaired in *GDI1*-null mice, while synaptic vesicle dynamics was also affected.^490^ Moreover, the deletion of *GDI* likely affected neuronal vesicle trafficking, thereby altering short-term synaptic plasticity and causing short-term memory deficits.^496^ Conditional and neurone specific deletion of *GDI1 (CamkII-Cre*^*+*^*-Gdi1*^*flox/Y*^ model), showed that the down regulation of αGDI in neurones in adult forebrain regions was sufficient to recapitulate the learning deficits previously shown in *Gdi1*-null mice.^497^

Astrocytes, however, also express αGDI and vesicle traffic in astrocytes is similarly impaired in the absence of *GDI1*.^498^ Proteomic analysis of astrocytes from astrocyte-specific knock-out *GDI1* animals^499^ revealed a significant change in the expression of genes responsible for glucose homoeostasis. Specifically, glycogen phosphorylase, the enzyme mediating glycogenolysis, was significantly decreased in *GDI1* deficient astrocytes Glycogen is in the brain predominantly, if not exclusively, expressed in astrocytes.^206^ In addition, expression of the mitochondrial isoform of phosphoenolpyruvate carboxykinase, involved in gluconeogenesis from non-carbohydrate carbon substrates such as pyruvate, lactate, glycerol and glucogenic amino acids, was significantly increased,^499^ indicating an elevated demand for free glucose through enhancing astrocytic glucose metabolism in *GDI1*-null mice. Indeed, measurements of noradrenaline (NA)-induced aerobic glycolysis by FRET nanosensors in individual astrocytes demonstrated an increased glucose utilisation, manifested as reduced NA-elevated free cytosolic glucose concentration, whereas the production of lactate by NA was unaltered.^499^ Measurements of NA-induced changes in cyclic adenosine monophosphate (cAMP) revealed an increased sensitivity of this second messenger to changes in extracellular lactate, indicating altered signalling landscape at the plasma membrane in relation to lactate homoeostasis. Behaviour in mice with astrocyte-specific *GDI1* deletion showed a selective and significant impairment in working memory, which was rescued by inhibiting glycolysis by 2-deoxy-D-glucose injection. These results indicate that astrocytes contribute to pathophysiology of ID and cognitive impairment.^499^

### Autistm spectrum disorders, ASD

Autism spectrum disorders (ASD; from Greek αυτός that means ‘being alone with yourself’) is a hypernym embracing the extended group of polyaetiological pathologies manifested by deviant social interactions, impaired language skills, and restrictive behaviours. The ASD-related nosological entities are many, yet they all seem to reflect an abnormal development of the CNS in pre- and early postnatal periods, linked to either genetic heritage or environmental insults.^500–502^ Glial contribution to the pathophysiology of ASD remains to be fully characterised.^503^ The transcriptome of brain samples from ASD patients demonstrated changes in expression of glial genes related to regulation of synaptogenesis and glial reactivity.^504^ Increased number of GFAP-positive astrocytes, oligodendrocytes and microglia were detected in the striatum, with no changes in glial numbers in the amygdala.^111^ Functionally, astrocytes derived from stem cells obtained from ASD patients and grafted into the rodent brain generated abnormal Ca^2+^ signals, which are arguably linked to impaired long-term potentiation and repetitive behaviours.^505^ Some evidence suggests that mutations in neuroligins expressed mainly in astrocytes and involved in regulation of synaptogenesis, can be related to ASD pathophysiology.^506^

A specific form of ASD, known as Rett syndrome (clinically manifested with microcephaly, loss of motor coordination, stereotypic hand wringing, ataxia, seizures, and sleep disturbances) is caused by a loss of function mutation of the methyl-CpG-binding protein 2 or *MECP2* gene, expressed in both neurones and neuroglia.^507^ Deletion of this gene from astrocytes decreases glutamate clearance and produces ASD-like behavioural phenotypes including aberrant locomotion and anxiety, whereas re-expression of wild type MECP2 gene in astrocytes alleviated these symptoms.^508,509^ Clinical presentation of Rett syndrome often includes aberrant breathing patterns, which can be mimicked in mice by astrocyte-specific deletion of *MECP2*,^510^ whereas re-introduction of the gene rescued abnormal breathing.^511^ A most common single-gene form of ASD is the fragile X syndrome, also known as Martin–Bell, or Escalante’s syndrome. Fragile X syndrome is caused by mutations in *FMR1* gene (which encodes fragile X mental retardation protein, FMRP) expressed in both neurones and astrocytes. Deletion of this gene in mice leads to the decrease in synaptic coverage by astrocytic leaflets, which translates into reduced densities of glutamatergic synapses.^512^ Deficits of astrocytic support of synaptogenesis can also be linked to a decrease in secretion of astrocyte-specific synaptogenic molecules such as hevin, SPARC proteins and thrombospondin-1.^513,514^ Astrocytes from *FMR1* knockout mouse model of fragile X syndrome were characterised by an enhanced secretion pf interleukin-6 and tenascin C, as well as by increased purinergic signalling.^515^

### Conclusions

Neurodevelopmental disorders are primarily associated with aberrant differentiation of neural cells. Notably, a premature shift of neural stem cells away from neuronogenesis and to gliogenesis seems to play a leading role in several syndromes. Furthermore, functional deficiencies of astrocytes promote neuronal damage, thus contributing to the cognitive deficits.

## Neurodegenerative diseases

### Astroglial decline in the ageing brain

Ageing is the main risk factor for many diseases, including an extended spectrum of neurodegenerative disorders leading to senile dementia. Conceptually, ageing reduces the functional capacity of all organs and systems, ultimately weakening the whole organism, reducing its adaptability, wearing out its defensive systems and bringing it to death through age-dependent diseases. In the process of physiological ageing, both systemic and tissue-specific systems show functional downfalls, which, when projected to the brain, translate into weakening defence and metabolic strain resulting from age-dependent declines of the cardio-vascular system and overall decreases in metabolism. The nervous system withstands ageing better than other organs and systems (a phenomenon recognised long ago^516^), which arguably reflects the high plastic potential allowing constant remodelling of the nervous tissue (the process which underlines life-long learning and adaptation) and high defensive and regenerative potential of neuroglia defining nervous system resilience and regeneration mending various insults accumulating during life-span.^115,517^ Indeed, even though human intelligence remains high long after 5^th^ decade of life, other systems show substantial decline in the physical capacities of youth.

Neuroglial changes in physiological brain ageing are relatively subtle, with white matter being the most affected: in healthy old age, white matter volume is decreased by ~ 10%, whereas the grey matter is reduced by a mere 3%.^518^ This reflects age-dependent decrease in myelin, linked to a decrease in the number of oligodendrocytes and their precursors.^519,520^ Microglia in aged human brain show substantial morphofunctional deterioration with ~ 40% of all microglial cells becoming morphologically dystrophic and functionally asthenic.^521,522^ Likewise ageing astrocytes became atrophic, thus reducing their homoeostasis and defensive support.^115^ These changes in neuroglia define the susceptibility of the brain to pathology and hence glial performance defines physiological (with cognitive preservation) versus pathological (cognitive decline) brain ageing.

Studying the transcriptome of the ageing brain revealed substantially more prominent changes in neuroglia, and in astroglia in particular, when compared to neurones. Transcriptome profiling of human post-mortem tissues (obtained from people aged between 16 and 102 years) found prominent and complex changes in gene expression in oligodendrocytes and astrocytes, whereas the neuronal transcriptome remained more or less undisturbed.^523^ Analysis of gene expression in cortical astrocytes from old mice identified an increase in genes linked to an immune response with a decrease in expression of GFAP and genes related to neuroprotection and neuronal support,^524^ and the prominent up-regulation of genes contributing to synapse elimination in astrocytes from aged hippocampus and cerebellum.^151^ Single-cell RNA sequencing of ~50,000 transcriptomes from young (3 - 4 months) and old (21–23 months) mice demonstrated substantial regional heterogeneity of age-dependent changes indicating that ageing of neurones and glia may develop through distinct molecular pathways.^525^

Age-dependent changes of astrocytic morphology have been understudied, with some controversial observations reported when only the morphology of GFAP-stained profiles was analysed, which can easily be misinterpreted. Using combinatorial approaches, total numbers of astrocytes seem not to change in physiological ageing neither in humans nor in rodents.^71,115,526^ Expression of GFAP is generally increased with brain ageing, which was regarded as a sign of widespread astroglial reactivity and age-dependent neuroinflammation.^527,528^ At the same time, morphometric analysis of GFAP-positive profiles delivered contradictory results, as both increases and decreases in size and complexity of GFAP-positive astrocytic profiles in ageing were reported (see ref. ^115^ for details). Golgi staining of aged astrocytes did not show major morphological changes^529^, whereas age-dependent changes in astrocytes stained with antibodies against S100B and glutamine synthetase are characterised by complex region-dependent changes ranging from atrophy to hypertrophy.^530^ In marmoset, GFAP-positive astrocytic profiles showed remarkable atrophy in advanced ages.^531^

**Fig. 12:**
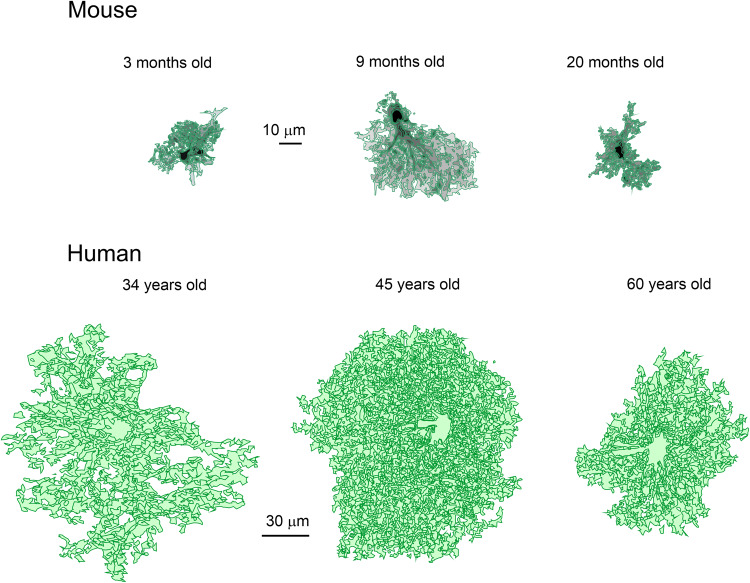
Astrocytic atrophy in ageing. Upper panel shows 3D reconstructions of Alexa 594 filled cortical astrocytes of mice of different ages. Reproduced from ref. ^114^ Lower panel shows 3D of Alexa 594 filled astrocytes from the cortex of human patients (tissue obtained during neurosurgery) of different ages. Reproduced from ref. ^532^

More accurate studies of morphology of astrocytes in aged mice in situ using intracellular injections of fluorescent dye Alexa Fluor 594 with subsequent two-photon imaging and 3D reconstruction identified a substantial reduction of astrocytic size and complexity with particular degradation of perisynaptic leaflets (Fig. 12).^114^ Very similar atrophic changes were found in human tissues obtained during surgery: again, substantial decreases in size, complexity and territorial domains of protoplasmic cortical astrocytes were documented (Fig. 12, ref. ^532^). Reduced astrocytic presence impaired glutamate clearance and K^+^ buffering thus affecting synaptic plasticity.^114^ Of note, supplying the old brains with young of stem cell-derived astrocytes in experimental rodents was beneficial, once more confirming the fundamental role of astrocytic homoeostatic support in brain ageing.^533^

**Fig. 13:**
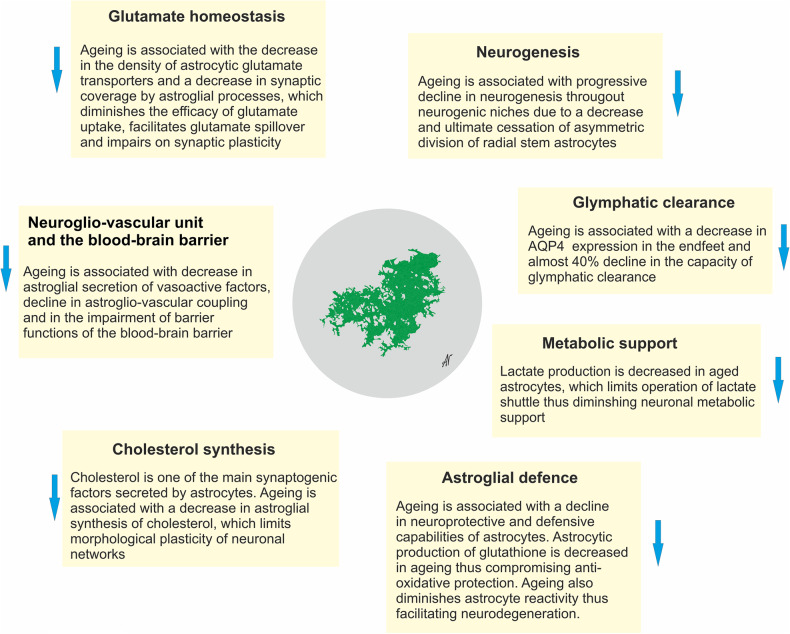
Decline in astrocytic functions in ageing

Functionally aged astrocytes retain their receptors and capacity of generating Ca^2+^ signalling in response to neurochemical stimulation.^534,535^ Resting membrane of aged astrocytes remains highly hyperpolarised (~−80 mV), the input resistance increases somewhat with age, thus reflecting morphological shrinkage.^114^ The density of AMPA, NMDA and P2X receptors as well as the density of plasmalemmal glutamate transporter currents demonstrates bell-shaped age dependency, with maximal densities observed in adult animals, whereas ageing is accompanied by a 2-3 fold decrease of density of respective ion currents.^534^ Ageing seems to affect astrocytic spontaneous Ca^2+^ oscillations, which occur ~20 times more frequently in 20 months old (aged) mice when compared to young adult mice of 2.5 months of age.^536^ Astrocytic syncytial coupling is also decreased with age, probably reflecting age-dependent down-regulation of connexins expression.^114,537^ Overall, fundamental physiological properties of astrocytes seem to reflect generalised atrophy of these cells in ageing. All major astroglia functions decline with age (Fig. 13). This decline arguably represents the principal mechanism for age-dependent neurological disorders. Glial paralysis opens the gate for neurodegeneration and other diseases of old age.

Homoeostasis of major neurotransmitters, which is the most fundamental function of astroglial cells, is impaired in old nervous tissue. Ageing of the brain is paralleled by an increase of the glutamate to glutamine ratio indicating abnormal operation of the glutamate (GABA)-glutamine shuttle.^538^ Aged astrocytes indeed express fewer glutamate transporters, resulting in age-dependent decreases in glutamate clearance.^539^ Glutamate transporter currents, which are a direct measure of the glutamate transport, decrease to ~15% of their level in young adult astrocytes.^534^ Astrocytic catabolism of catecholamines is also affected by ageing. Astrocytic expression of MAO-B rises 2–3 times in old nervous tissue,^540^ which limits catecholamine bioavailability and may contribute to neurodegeneration.^541,542^ Finally, aged astrocytes were reported to increase synthesis and release of GABA, which may impair excitation-inhibition balance on the neuronal networks.^543,544^

Ageing also affects astrocytic endfeet and glia limitans perivascularis. Ageing is associated with increased thickness of capillary walls, basement membranes, and astroglial endfeet vascular coverage.^545^ In the ageing brain, AQP4 migrates away from endfeet, which reduces operational capacity of the glymphatic system by ~40%.^546^ Morphological atrophy of astrocytes also reduces their presence in the neuropil, which leads to an increase of extracellular diffusion channels and hence to an increase in mean diffusivity of the grey matter, detected in elderly humans with diffusion tensor imaging.^547^ Metabolic decline of aged astrocytes is also well documented.^548,549^ In addition, ageing may affect astrocyte-dependent neurotransmitter clearance, thus further limiting neuronal support.^550^ Old astrocytes substantially down-regulate synthesis of cholesterol due to a decreased expression of the main cholesterol synthesising enzyme HMG-CoA reductase,^151^ which limits synaptogenesis and membrane repair. Ageing is also associated with a prominent decrease in the neurogenic potential reflecting functional asthenia of stem radial astrocytes.^551–553^ Finally ageing impairs the ability of astrocytes to mount protective reactive astrogliosis^120^ thus limiting brain protection against pathological insults.

To summarise, ageing is accompanied by significant decline in astrocytic haemostatic and neuroprotective capacities, which may directly affect nervous tissue and increase it vulnerability to pathological attacks and neurodegeneration.

### Amyotrophic lateral sclerosis, ALS

Amyotrophic lateral sclerosis is a malignant and rapid degeneration of upper and lower motor neurones located in the cortex, brain stem and spinal cord. Clinically, ALS presents itself as a progressive ascending paralysis and muscle atrophy leading ultimately to respiratory failure and death.^554^ Astrocyte pathology is a fundamental, if not leading, factor in neuronal death: astrocytes in ALS lose their capability to sustain neurones, to clear glutamate and they some aberrant astrocytic forms are generated, which may cause direct damage.^555–557^ In addition, astrocytic vesicular traffic in ALS context is enhanced, which is linked to aberrant Ca^2+^ signalling and homoeostasis.^558^ Although most ALS is sporadic, several familial genetic forms also exist and have provided animal models. Studies of a mouse model of familial ALS that express human mutant superoxide dismutase 1 (SOD1^G93A^) support a primary role of astrocytes. Expression of mutant SOD1 in neurones did not result in ALS symptoms.^559^ In contrast, silencing of this gene in astrocytes in global SOD1 expressing mice with ALS-like symptoms, arrested pathological progression.^560,561^ Similarly, grafting astrocytes hosting SOD1^G93A^ mutant into the spinal cord of the healthy mice promoted motor neurones demise resulting in ALS symptoms.^562^ At the same time transplanting healthy astrocytes into the spinal cord of globally SOD1^G93A^ mutant expressing rats delayed pathological progression.^563^

At least three major pathological astrocytic phenotypes have been identified in ALS: these are degenerating/atrophic, reactive and aberrant morphotypes. Functionally all of these phenotypes are characterised by loss of several homoeostatic molecules, which decreases neuronal support and neuroprotection ultimately resulting in neuronal death.

Pathological astrocytes, when mixed with healthy neurones, can, on their own precipitate neuronal death, again indicating a leading role of astrocytes in pathophysiology of ALS. Astrocytes derived from stem cells generated from ALS patients cannot sustain neurones in co-culture; instead, they decrease neuronal survival.^564^ Likewise, primary astrocytes prepared from ALS post-mortem samples induced necroptosis of neurones in co-culture,^565^ and transplantation of diseased astrocytes into the spinal cord triggered motor deficits and promoted motor neurones degeneration.^566^ Neurotoxicity is likely to be associated with aberrant proliferating astrocytes, which express astrocytic and microglial markers, and are characterised by the presence of lipid droplets and many secretary vesicles. Aberrant astrocytes cannot provide homoeostatic support, but instead generate reactive oxygen species that may mediate neurotoxicity.^139,158,557,567^

Atrophic and reactive astrocytes in ALS demonstrate profound loss of homoeostatic and supportive function. One of the leading mechanisms of astrocyte-associated neurotoxicity is associated with severe decrease in the expression of glutamate transporters, the presence of which is reduced, in ALS patients, to only ~10% of the levels in healthy subjects.^144^ Comparable decline in glutamate transporters is observed in the SOD1^G93A^ mouse model of ALS.^568,569^ Deceased expression of astrocytic glutamate transporters was also detected in SOD1^G93A^ mice,^568,569^ whereas transgenic overexpression of EAAT2^570^ or pharmacological stimulation of its expression with riluzole^571,572^ delayed progression of experimental APS. Astrocytes in ALS show many signs of functional decline, for example they demonstrate abnormal Ca^2+^ signalling,^573^ impaired secretion of neuroprotective glial-derived neurotrophic factor,^574^ as well as reduced lactate production.^575^

### Fronto-temporal dementia, FTD

The role and contribution of astroglia to FTD remains largely unexplored. It seems however, (at least based on a limited number of studies) that degeneration, loss of function, dysregulated metabolism, impaired second messenger signalling and death of astrocytes are predominant pathophysiological manifestations.^576,577^ Astrocytes in FTD up-regulate apoptotic markers and show signs of apoptotic death^577,578^; moreover the prevalence of apoptotic astrocytes seems to correlate with the severity of the dementia.^579^ Clasmatodendrosis of astrocytes was observed in the majority of post-mortem samples from FTD patients.^121^ Mutations in the progranulin gene, *GRN*, which cause a genetic familial FTD, downregulate expression of EAAT2 astrocyte glutamate transporter, which mediates synaptic degeneration.^143^

### Alzheimer’s disease

Alzheimer’s disease is a complex pathology that appears in early onset familial forms (~1–2% of all cases; hereditary disease linked to mutated genes encoding amyloid precursor protein APP, presenilins1 and 2, and tau protein) and late onset sporadic forms for which age is the main risk factor.^580,581^ Clinical progression of AD is defined first by massive synapse loss and subsequently by neuronal death, which ultimately results in profound brain atrophy and is clinically manifested by severe dementia. Senile plaques (extracellular depositions) and neuronal tangles (intracellular accumulation of misphosphorylated tau protein), are the main histopathological hallmarks of the AD, critical for post-mortem diagnosis. The once widely accepted primary role for β-amyloid in sporadic AD (known as the amyloid cascade hypothesis) is currently criticised as not being the sole cause of AD, but nevertheless, amyloid accumulation is clearly a part of AD pathology and evokes multifaceted glial responses.^582–584^

Astrocytic changes in AD are complex, with disease stage and brain region specificity and include reactivity, degeneration and clasmatodendrotic death, and atrophy with loss of function.^34,585^ Astrocytic reactivity in AD is arguably triggered by β-amyloid deposits; although at the advanced stages of the disease the dying neurones and compromised blood-brain barrier may act as instigators. In post-mortem specimens from AD victims, reactive, hypertrophic astrocytes as well as reactive microglia surround senile plaques, thus creating a protective barrier.^586,587^ Similarly, reactive astrocytes are associated with β-amyloid deposits in the brains of various transgenic AD models (most of which are models of hyperamyloidosis), although sometimes reactive remodelling of astrocytes precedes formation of β-amyloid plaques.^588,589^ Astrocytic reactivity in the vicinity of senile plaques is, as a rule, of anisotropic, non-proliferative, mild variety; territorial domains of astrocytes surrounding amyloid plaques do not overlap, and astrocytes never form a barrier resembling that around traumatic or ischaemic lesions. There are also no indications for fibrotic changes in the nervous tissue of AD patients and AD animal models. Reactive astrocytes in AD are characterised by up-regulated GFAP expression, enlarged soma and thickened main processes.^71,590,591^ At the same time neither GFAP levels nor astrocytic reactivity correlates with the severity of pathology^592^; and no significant difference in GFAP was found in the brains of demented and cognitively preserved people of advanced age.^593^ Reactive astrocytes play predominantly protective role, and inhibition of reactivity exacerbates pathology in AD animal models.^594^ Astrocytic reactivity is most likely triggered by β-amyloid^595^ and subsequent Ca^2+^ release from the ER.^596^ Notably, memory loss in the PS2APP mouse AD model has been linked to astrocyte Ca^2+^ hypoactivity due to reduced expression by astrocytes of the calcium sensor, STIM1; and overexpression of STIM1 selectively in astrocytes fully recovers astrocyte Ca^2+^ activity and synaptic plasticity in this model.^597^

Clasmatodendrotic astrocytes were found both in human post-mortem samples^122,598^ and in APP-SweDI (Swedish-Dutch-Iowa mutation of APP) expressing mice.^599^ The interlaminar astrocytes (which populate brains of high primates and are not present in other species^32,33^) are highly vulnerable to AD pathology: at the advanced stages of the disease interlaminar astrocytes disappear,^600^ although how this impacts on the disease pathophysiology remains unknown. Protoplasmic astrocytes with atrophic morphology were found in triple-transgenic (3xTG-AD; harbour mutant APP, PS1 and tau) and PDAPP-J20 (expressing Swedish-Indiana APP mutations). These astrocytes, characterised by reduced somata volume, thinner and less complex processes, are present in cortical regions and in the hippocampus.^71,533,601–604^ Morphological atrophy of astrocytes develops in a brain-region-linked fashion, starting first in the entorhinal and prefrontal cortices (which are, incidentally, the most vulnerable to AD) and then spreading to hippocampus.^116,605,606^ Reminiscent of the animal models, astrocytes differentiated from induced pluripotent stem cells obtained from patients with both familial and sporadic forms of AD are smaller and less complex compared to astrocytes form healthy controls; in addition, these ‘diseased’ astrocytes demonstrate abnormal localisation of key astrocytic markers.^607^ In functional studies, astrocytes differentiated from stem cells obtained from familial AD showed aberrant Ca^2+^ signalling,^608,609^ increased production of reactive oxygen species, reduced lactate supply, compromised neuroprotection, and inability to support neurones in co-cultures.^610,611^ Furthermore, APOE4-expressing astrocytes differentiated from stem cells could not internalise β-amyloid and had abnormal cholesterol accumulation.^612^ Astrocytic dystrophy and glucose hypometabolism were also observed in brain imaging of AD patients.^595^ Transcriptome analyses suggest both gain and loss of functions in astrocytes in AD mouse models, with down-regulation of genes associated with neuronal support and upregulation of inflammation related genes.^613,614^

Astrocytes in AD animal models, as well as astrocytes differentiated from stem cells isolated from AD patients, are characterised by decreased energy metabolism, decreased glucose uptake and glucose consumption.^615,616^ Deficient glycolysis limits production of lactate for neuronal support and L-serine, an obligatory precursor of D-serine, which neurones secrete to sustain synaptic plasticity.^617^ Exposure of astrocytes to β-amyloid down-regulates expression of glutamine synthetase,^618^ and in animal models, glutamine synthetase expression is reduced in astrocytes surrounding β-amyloid plaques.^619^ Likewise, the activity of glutamine synthetase is profoundly decreased in the brain tissue of AD patients due to protein oxidation.^620^ Astrocytic production of glutamine and associated glutamate-glutamine shuttle operation are reduced in AD, affecting synaptic transmission as well as detoxification of ammonium.^447^ Furthermore, astrocytic atrophy and reduced presence of astrocytes around synapses affects synaptic maintenance, glutamate homoeostasis, and K^+^ buffering thus affecting neuronal excitability and limiting synaptic plasticity.^116^ Astrocytic asthenia is also manifested by the limited reactivity in the most vulnerable brain regions such as entorhinal and prefrontal cortices in which astrocytes do not mount a defence response in the presence of β-amyloid depositions.^116,605,606^ Similarly, astrocytic reactivity seems to fade at the advanced stages of AD in human patients.^621^

Astrocytic remodelling in AD also includes an increased synthesis and secretion of GABA: high GABA concentrations were found in astrocytes in animal models and in AD patients.^154,543,544^ Increased GABA synthesis reflects up-regulation of glutamic acid decarboxylase GAD67 and monoaminoxidase-B (MAO-B), the latter producing GABA from putrescine.^154^ Increase in expression of MAO-B is arguably linked to an aberrant activity of the urea cycle linked β-amyloid.^622^ Secretion of GABA from astrocytes, likely mediated by Best1 anion channels,^623^ may increase tonic inhibition thus counteracting neuronal hyperexcitability characteristic for AD.^624^ In particular, astrocyte-derived released GABA activates neuronal GABA_A_ and GABA_B_ receptors, which, in turn, inhibit neuronal activity. Thus, GABA from reactive astrocytes diminishes the spike probability of the perforant-path-to-dentate-granule-cell synapse, leading to impairment in synaptic plasticity and memory function.^155^ The by-product of the putrescine catabolism is hydrogen peroxide, which is released from reactive astrocytes in the AD thus adding to the damage of the nervous tissue. Treatment of animals with excessive β-amyloidosis with newly developed reversible MAO-B inhibitor KDS2010 or the potent H_2_O_2_ scavenger AAD-2004 ameliorated neurodegeneration.^155^ At the molecular levels astrocytic switch to putrescine catabolism and GABA synthesis is linked to the urea cycle expressed in astrocytes and generally responsible for detoxification of brain ammonium. Astrocytes in AD model animals demonstrated up-regulated expression of genes (CPS1, OCT, ASL, ARG1, and ODC1) and metabolites (aspartate, ammonia, urea, putrescine, and GABA) of the urea cycle. In healthy CNS tissue, urea metabolism is non-cyclic, whereas it becomes fully cyclic upon exposure to β-amyloid. Astrocytic uptake of β-amyloid leads to it autophagic degradation, entrance of excess aspartate and ammonia into the urea cycle, increased putrescine and GABA production, with increased H_2_O_2_ production and neuronal damage leading to cognitive deficits. Inhibition or down-regulation of ODC1 breaks this vicious cycle and promotes astrocytic detoxification of β-amyloid.^625^ This demonstrates how physiologically relevant metabolic pathways employed by astrocytes for maintaining the homoeostasis of the nervous tissue may acquire detrimental proportions under conditions of excessive or prolonged stress.

**Fig. 14:**
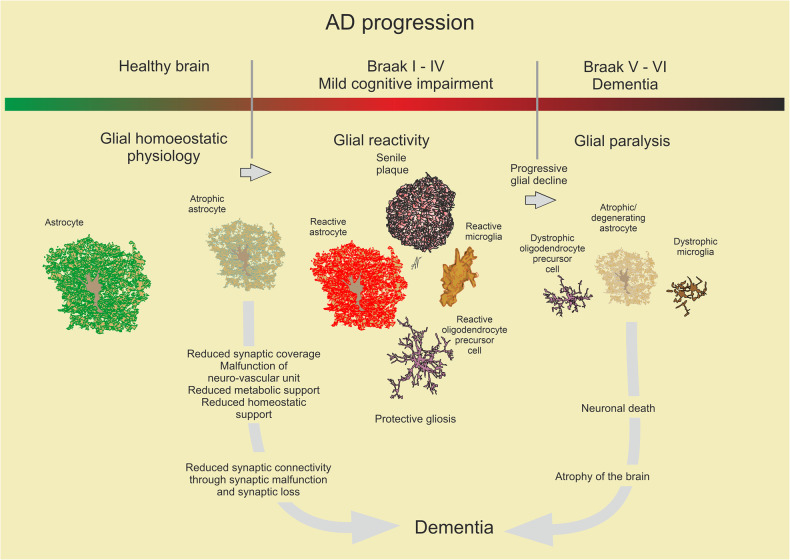
Glial reactivity, decline and paralysis define the pathophysiology of AD. At the prodromal and early stages of the disease astrocytes display atrophic morphology possibly indicating limited homoeostatic support, which may lead to the early synaptic malfunction. After emergence of the plaques reactive astrocytes and microglia surround β-amyloid depositions to protect the nervous tissue. Progressive decline in glial neuroprotection and homoeostatic support culminates in glial paralysis which permits neuronal death and brain atrophy manifested in dementia

All in all, astrocytic responses in AD are complex and disease stage-specific: in the early, compensated phase of AD (manifested by mild cognitive impairment) astrocytes, together with other neuroglia become reactive and arguably protect brain tissue. At advanced AD stages, glial paralysis contributes to brain tissue atrophy and clinical dementia (Fig. 14).

#### Astrocytes as targets for AD management and therapy

At present is seems unlikely that a single magic bullet will be found to cure AD or other neurodegenerative diseases leading to dementia. Nevertheless, various approaches that may help sustain cognitive resilience during aging are emerging. For example, lifestyle modifications in the widest possible context can prolong cognitive longevity; in particular dieting, intellectual and physical activity, as well as social engagement were shown to delay or even improve age-dependent cognitive impairments.^626–628^ Experimental evidence is accumulating that astrocytes readily respond to lifestyle modifications, and may be instrumental in conferring clinical benefits. For example, exposure of mouse AD model to either enriched environment or voluntary physical activity, or combination of both reversed astrocytic atrophy and decreased β-amyloid load.^92,603^ Environmental stimulation and voluntary exercise also restores neurogenesis that is severely inhibited in AD mice.^552,629^ Dieting is another easily modifiable lifestyle factor. Caloric restriction in particular may increase cognitive resilience and sustain cognitive longevity.^630^ Caloric restriction regimen translates into increased astrocytic complexity, size of astrocytic territorial domains and volume of perisynaptic leaflets, paralleled with an improved glutamate clearance and K^+^ buffering, all improving synaptic plasticity.^631^ Special diets with high intake polyunsaturated fatty acid 2-hydroxy-docosahexaenoic were also shown to rescue astroglial atrophy, restore adult neurogenesis and improve cognitive performance in 5xTG AD mouse model.^632^

Astrocytes are primary targets for noradrenergic innervation^633^ that are provided by widespread projections of neurones located in locus coeruleus and are critical for cognitive functions. Neurones of this nucleus are particularly vulnerable to ageing and neurodegeneration and locus coeruleus is arguably the first location being affected in AD. Enhancing bioavailability of noradrenaline or adrenergic responsiveness of astrocytes could be a valid therapeutic strategy.^541,542^ This can be achieved by inhibiting astrocytic MAO-B, and indeed deperenil (aka selegiline) showed some efficacy in improving memory and clinical progression of AD.^634^ Inhibitors of MAO-B can also reduce hydrogen peroxide production linked to the putrescine catabolism. The transcranial direct current stimulation, which improves cognitive symptoms in AD patients, is mediated through α_1_-adreniceptors-mediated massive Ca^2+^ signalling in cortical astrocytes.^635^ Further exploration of such potential mechanisms that involve astrocyte is warranted.

### Parkinson’s disease

Parkinson’s disease named after James Parkinson who provided the first description of this disorder,^636^ is characterised by progressive degeneration of dopaminergic midbrain neurones of the brainstem. Clinically the disease is manifested after ~70% of dopaminergic neurones die.^637^

Both astrocytic reactivity and astrocytic atrophy with loss of function are observed in affected brain regions in PD. Astrocytic reactivity,^638,639^ might be of secondary nature as a response to neuronal damage, especially in toxic animal models of PD. Reactive astrocytes in the context of PD increase production of ROS and decrease antioxidative protection, which may translate into neuronal damage.^640,641^ At the same time astrocytes in brain samples and in organoids made from iPSCs-differentiated astrocytes form Parkin-mutation familial PD had a much decreased GFAP expression compared to controls.^642^ Astrocytes obtained form stem cells isolated from patients with PD linked to LRRK2/dardarin mutation showed prominent morphological atrophy,^113^ as well as decreased expression of glutamate transporters,^643^ and prominent mitochondrial deficiencies.^113,644^ Astrocyte-selective expression of PD related A53T mutant α-synuclein causes severe downregulation of glutamate transporters expression, resulting in neuronal damage, aberrant microgliosis and paralysis.^645^ Protoplasmic (but not fibrous) astrocytes protect against α-synuclein toxicity by removing this latter through endocytosis,^646,647^ and hence astrocytic atrophy reduces neuroprotection.

Mitochondrial insufficiency in astrocytes is a hallmark of PD, leading to a profound deterioration of astrocytic homoeostatic, supportive, and protective capabilities.^648^ Maintenance of metabolic support and mitochondria pool of striatal neurones seems to be one of the astrocytic functions which possibly define the pathophysiology of PD. In particular astrocytes can be a central element in maintaining mitochondrial function of dopaminergic neurones thorough transmitophagy, the process when astrocytes receive and degrade damaged neuronal mitochondria,^649^ or even supply healthy mitochondria.^650^ Astrocyte-neuronal mitochondrial exchange, which alleviated neuronal damage was recently demonstrated in the co-cultures systems^651,652^; in particular mitochondrial donation rescued dopaminergic neurones.^652^

### Huntington’s disease

Huntington’s disease (HD, or Huntington’s chorea; named after George Huntington who was the first to describe it^653^) is caused by a single dominant allele of the huntingtin gene containing an expanded number of CAG repeats; the disease develops when the number of repeats exceeds 40.^654^ Of note, astrocyte-specific deletion of mutant huntingtin in mice globally expressing this gene eased disease symptoms and delayed its progression, indicating a role for astrocytes in the pathophysiology of the disease.^655^ Astrocytes in HD undergo morphological atrophy and loss of many homoeostatic functions. In animal models of HD, atrophic astrocytes in striatum retract their leaflets from cortico-striatal synapses, which are known to be affected in the early HD.^656^ Similar atrophy was found in human astrocytes expressing mutant huntingtin and transplanted into the corpus callosum of mice.^657^ Astrocytic loss of function includes compromised K^+^ buffering,^128^ glutamate transport and Ca^2+^ signalling.^117^ Significant decrease in expression of K_ir_4.1 channels in striatal astrocytes was also detected in human tissue.^658^ Likewise, expression of glutamate transporters is reduced in astrocytes from human tissue and animal models of HD^112,659^; in particular astrocyte-selective expression of mutated huntingtin with 160 GAG repeats triggered profound down-regulation of EAAT2 and HD-like phenotype in mice.^660^

### Astrotauopathies

**Fig. 15:**
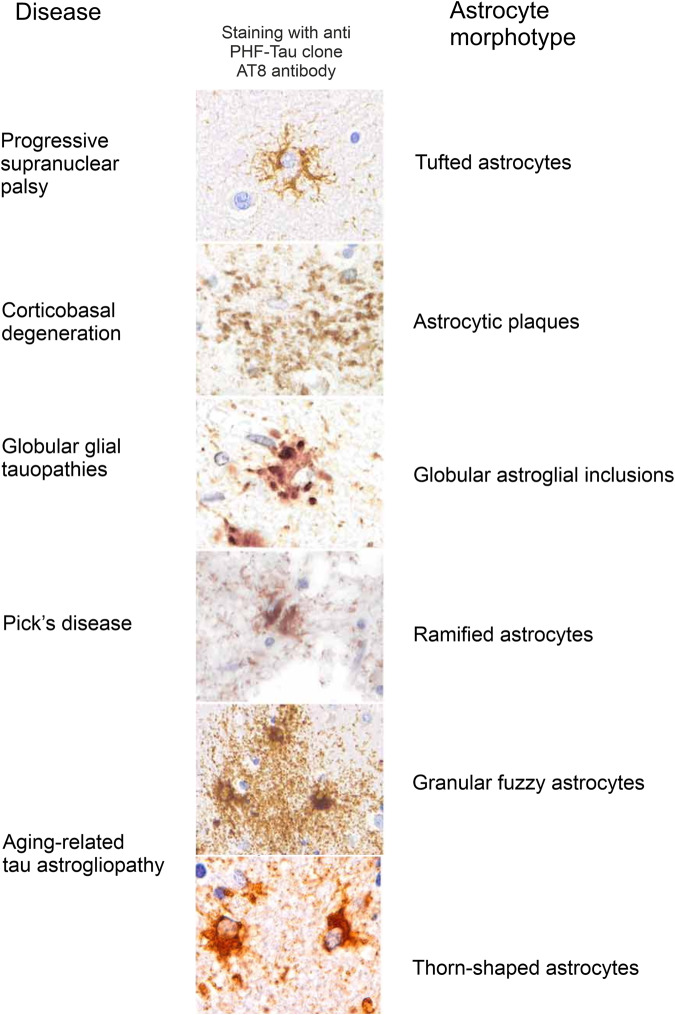
Histopathology of astrocytes in astrotauopathies. See text for explanation. Modified, and reproduced from the images kindly provided by Professor Gabor Kovacs, Toronto University

Astrotauopathies are a distinct class of neurodegenerative diseases caused by an abnormal accumulation of tau exclusively in astrocytes.^661,662^ Astrotauopathies result in astrocytes with various morphotypes classified as (i) astrocytic plaques, (ii) tufted astrocytes, (iii) ramified astrocytes, (iv) globular astroglial inclusions, (v) thorn-shaped astrocytes and (vi) granular/fuzzy astrocytes (Fig. 15,^661^). Astrotauopathies drive the pathophysiology of progressive supranuclear palsy, corticobasal degeneration, Picks disease, argyrophilic grain disease, globular glial tauopathies, and ageing-related tau astrogliopathy (ARTAG).

Tufted astrocytes, characterised by accumulation of tau in proximal astrocytic processes, represent the histopathological hallmark of progressive supranuclear palsy, a rare neurodegenerative disorder with progressive impairment of balance, walking, eye movement, muscular rigidity, dysarthria, and dysphagia.^663,664^ Corticobasal degeneration is a primary astrocytopathy characterised by the accumulation of astrocytic plaques. These are visualised as fuzzy short argyrophilic processes arranged annularly with fine collaterals at vertical or sharp angles with tau is accumulated mainly at the distal parts of enlarged astrocytic processes.^664,665^ Clinically, corticobasal degeneration is manifested by movement deficits, impaired swallowing, speech, and memory. As the disease progresses, tau spreads to neurones causing cell death.

Another example of a primary astrocytopathy is the frontotemporal lobar degeneration or Picks dementia, leading to progressive loss of memory, primary progressive aphasia, and social misbehaviour. The onset of the disease is associated with massive dystrophy and death of astrocytes, which directly correlate to the severity of the disease.^579,666^ Ramified astrocytes with tau depositions in soma and in processes are the histopathological hallmark of the Pick’s disease.^667^ Global primary tauopathies, characterised by widespread 4-repeat tau inclusions in astrocytes and oligodendrocytes result in dementia dues to neurodegeneration in frontal and temporal lobes.^661,668^ Finally, ARTAG, which represents a spectrum of age-dependent dementias caused by tau accumulation in astrocytes. Pathological astrocytic morphotypes are (i) thorn-shaped astrocytes with tau inclusions in soma and proximal processes, and in the subpial and perivascular endfeet and (ii) granular-fuzzy astrocytes with fine granular tau inclusions mainly in the perinuclear region.^662^ This latter type of astrocyte was also detected in argyrophilic grain disease, in AD and HD.^662^

### Conclusions

The contribution of neuroglial cells to pathophysiology of neurodegenerative diseases is complex and heterogeneous with substantial disease and disease-stage stage specificity. Aberrant and diseased astrocytes promote death of motor neurones in amyotrophic lateral sclerosis, whereas loss of astrocyte homoeostatic support contributes to neuronal malfunction and demise in AD, PD, and HD.

## Future perspectives: astrocyte targets as a frontier for new therapies for neurological disorders

Astroglia (as well as all neuroglial cells) provide for comprehensive support of the CNS; together with other neuroglia, astroglia are indispensable elements of the nervous tissue maintaining its normal function in health and guarding it against disease. The pathophysiology of astroglia is complex, highly heterogeneous and mutable: multiple pathology-associated phenotypes may co-exist and emerge and disappear during the progression of a neuropathology. In the absence of detrimental genetic mutations or polymorphisms, any lesion of the brain triggers astroglia responses that are in the first instance aimed at the preservation and/or restoration of homoeostasis. Profound lesions resulting in the death of parenchymal cells and inflammation instigate reactive astrogliosis that forms a border between damaged non-neural lesion core and surrounding neural tissue. This border is of paramount importance for the survival of adjacent neural tissue and is ultimately required for post-lesional tissue regeneration. Most chronic diseases of the CNS are associated with loss of function of astrocytes, and such loss of function is emerging as a leading mechanism of disorder-related neurological dysfunctions ranging from mild changes in synapse regulation to potential overt neurotoxicity in extreme cases. Contrary to a popular belief, astrocytes do not, through a specific pre-programmed gain of function, generate a specific toxin(s) or adopt a specific toxic phenotype proposed to be common across many diseases that may represent a ‘universal’ therapeutic target. There is as yet no rigorous evidence for this. Instead, the disease and context specific loss or disruption of astrocyte functions can result in the accumulation of various potentially toxic metabolites or in the loss of support functions for neurones or synapses or in (the deadliest) combination of both. Notably, astrocyte loss of functions can lead to elaboration of potentially toxic molecules such as ROS or certain lipids and this may be mistaken for a ‘purposeful’ gain of detrimental function. Astrocytic functional paralysis translates into neuronal damage or death because neurones themselves are incapable of preserving tissue homoeostasis. Thus, understanding, and counteracting disease-associated astrocyte loss of functions should be a major therapeutic goal. As yet, we are not in a possession of astrocyte-specific therapies or drugs; nonetheless we know that many medicines or lifestyle changes do affect astroglia. Commonly positive outcomes are linked to increases in astrocyte presence and or up-regulation of key homoeostatic cascades (the glutamate transporters being most singular targets). In this article we have highlighted many different astrocyte-associated molecules that are emerging as potential therapeutic targets in different disorder contexts. The extensive breadth of these different molecules highlights the diversity of astrocyte reactivity not only across disorders, but within the same disorder at different timepoints or in different CNS locations. This observation in turn emphasises the importance that therapies aimed at modulating astrocyte reactivity will need to modulate specific molecules and specific aspects of reactivity in a disorder and context specific manner. The notion that astrocyte reactivity is somehow globally harmful per se and should therefore be blocked in its entirety is likely to do more harm than good and is no longer tenable. Notably, in addition to trying to block potential downstream detrimental effects, new therapies need to be designed that preserve and boost astrocytic defences and improve astrocytic homoeostasis; such therapies may turn the tide by ultimately developing pathophysiology-based treatments of CNS diseases.
